# Bacterial Extracellular Vesicles (BEVs) Derived From *Lactococcus lactis* as Multimodal Drug Delivery Platforms

**DOI:** 10.1155/ijbm/3141223

**Published:** 2025-12-31

**Authors:** Sushmita Das, Subrata Das, Subhadeep Gupta, Afruja Khan, Pradip Kumar Tarafdar, Amirul Islam Mallick

**Affiliations:** ^1^ Department of Biological Sciences, Indian Institute of Science Education and Research Kolkata, Mohanpur, Nadia, West Bengal, 741246, India, iiserkol.ac.in; ^2^ Department of Chemical Sciences, Indian Institute of Science Education and Research Kolkata, Mohanpur, Nadia, West Bengal, 741246, India, iiserkol.ac.in

**Keywords:** Antimicrobial and anticancer drug delivery, bacterial extracellular vesicles (BEVs), *Lactococcus lactis*

## Abstract

Advances in drug delivery technologies involve the extracellular vesicles as a promising bioactive drug delivery vehicle expected to improve targeted therapeutic delivery in pharmaceutical innovations. Recently, Gram‐negative bacterial extracellular vesicles (outer membrane vesicles or OMVs) have gained attention for their role in host–microbe interactions and potential in drug delivery. Bacterial extracellular vesicle (BEV) shedding is a conserved mechanism of intra‐ and interspecies communication, providing critical insights into host–microbe interactions. However, the biogenesis and compositional diversity of BEVs produced by Gram‐positive bacteria remain underexplored. Understanding the translational application potential of BEVs remains elusive due to the suboptimal isolation of BEVs and limited structural–functional characterization. A comprehensive study to develop BEVs as delivery vehicles will provide critical insights into the perspective of microbial–host interplay and illuminate the modulation of the drug delivery strategy. Here, using a food‐grade probiotic *Lactococcus lacti*s subsp. *cremoris* MG1363 (strain NZ9000), we demonstrated that perturbing peptidoglycan biosynthesis with ampicillin, which targets penicillin‐binding proteins (PBPs), significantly enhances BEV production. We further explored the interaction between BEVs and host cells through this optimized BEV biogenesis, revealing its cargo‐delivering capability. Furthermore, to understand the potential of BEVs as a multimodal drug delivery platform, we target multidrug‐resistant microbial pathogens and cancer cell proliferation with drug‐encapsulated BEVs. With a generally recognized as safe (GRAS) recognition of *L. lactis,* we demonstrated that drug‐loaded *L. lactis* BEVs can offer recognizable therapeutic effects. These findings highlight the versatile nature of *L. lactis* BEVs as stable, safe, natural nanocarriers capable of personalized cargo delivery with broad therapeutic applications.

**Summary**

• Developed an optimized enrichment protocol for the biogenesis of bacterial extracellular vesicles (BEVs) from *Lactococcus lactis*, a Gram‐positive probiotic bacterium.
• Characterized the morphological, functional, and biological attributes of BEVs, providing new insights into their properties
• Demonstrated the potential of BEVs as safe nanocarriers for drug delivery, highlighting their broad therapeutic applications.

## 1. Introduction

Advancements in drug delivery technologies have revolutionized pharmaceuticals by enhancing targeted therapy while minimizing unintended effects [1]. As therapeutic modalities have expanded to include nucleic acids, peptides, proteins, antibiotics, and antibodies, drug delivery systems have evolved to meet the challenges associated with safety, efficacy, and biocompatibility [2]. Among these advancements, bioinspired drug carriers have made significant strides. Notably, extracellular vesicles (EVs) have garnered attention because of their nanorange vesicular entity and ability to be shed naturally by various units of life, including eukaryotic and prokaryotic organisms [3–5]. In bacteria, these vesicles are classified as outer membrane vesicles (OMVs) if secreted by Gram‐negative bacteria, whereas vesicles released by Gram‐positive bacteria are named bacterial EVs (BEVs) [6]. The difference in nomenclature primarily stems from the distinct biogenesis processes involved in either the outer membrane of bacteria (for Gram‐negative bacteria) or the cell wall (for Gram‐positive bacteria). However, regardless of bacterial type, BEVs or OMVs can both carry diverse cargoes, including cellular metabolites, toxins, nucleic acids, and various subcellular components [7]. However, the biomedical application of BEVs as drug delivery vehicles faces challenges due to their low abundance, primarily because of their complex biogenesis [8]. To address this, we developed an optimized protocol for BEV enrichment by targeting peptidoglycan (PGN), a major component of bacterial cell walls that is a protective barrier against the external environment and physical extremes [9, 10].

We selected *Lactococcus lactis* as our model organism due to its nonpathogenic nature and designation as a generally recognized as safe (GRAS) bacterium. Given the well‐established role of bioengineered lactic acid bacteria (LAB) as efficient mucosal delivery platforms, we hypothesized that optimizing BEV production from *L. lactis* could unlock its multimodal applications. Various physiological factors, including pH, temperature, growth medium composition, and antibiotic exposure, have been shown to modulate EV biogenesis by influencing proteome dynamics and cell wall plasticity [11]. This study leveraged a subinhibitory concentration (< MIC_50_) of the third‐generation β‐lactam antibiotic ampicillin to hinder PGN synthesis, thereby enhancing BEV production.

Using this enrichment scheme, we investigated the functional properties of BEVs, focusing on their cellular uptake mechanisms through Förster resonance energy transfer (FRET) assays and in vitro endocytosis studies. Fluorophore‐labeled liposomes confirmed that BEVs undergo efficient membrane fusion, primarily via lipid mixing. Coincubation with murine J774.A1 macrophages and immortalized human cells (HeLa cells) cells further demonstrated that BEV lipids are highly fusogenic and can be internalized through dynamin‐dependent pathways.

Finally, we explored the potential of BEVs as drug carriers, demonstrating that drug‐loaded *L. lactis* BEVs effectively inhibit multidrug‐resistant pathogens and suppress the proliferation of cancer cells. While artificial nanocarriers have established safety and efficacy profiles, challenges such as high synthesis costs, limited shelf life, and uncertain biological fate remain significant hurdles. In contrast, probiotic‐derived BEVs offer superior safety, biocompatibility, and cost‐effectiveness. Our findings highlight the optimized BEV enrichment from *L. lactis*, which provides distinct advantages over conventional drug delivery systems [12].

Collectively, these results establish BEVs as promising nature‐inspired nanocarriers for clinical and biomedical applications, warranting further investigation into their large‐scale production and targeted therapeutic translation.

## 2. Results

### 2.1. Physicochemical Characterization of BEVs Derived From *L. lactis*

The secretion of BEVs from *L. lactis* was investigated to study the natural vesicle biogenesis process of *L. lactis* (Figure 1(a)). Field emission scanning electron microscopy (FESEM) analysis of the bacteria at two different growth phases (early log phase and late stationary phase) revealed the spontaneous nature of vesicle formation, predominantly occurring during the late exponential phase of bacterial growth (Figure 1(b): A‐B), identifying the “budding out” of BEVs from the surface of *L. lactis*. BEVs were isolated from the bacterial culture medium by ultracentrifugation. To check the morphological aspects, cryo‐transmission electron microscopy (TEM) (Figure 1(b): E) microscopy of isolated naturally secreted BEVs was conducted, which further confirmed the lipid‐bilayer vesicular structure of BEVs, where the lipid membrane was approximately 5–7 nm in length, resembling a standard lipid bilayer and exhibiting an indistinguishable size distribution profile of 20–100 nm. Similarly, the FESEM (Figure 1(b): C) and TEM (Figure 1(b): D) image analysis of isolated BEVs revealed a spherical vesicular morphology with a heterogeneous size distribution ranging from 10 to 100 nm. Furthermore, dynamic light scattering (DLS) analysis revealed that the average hydrodynamic diameter of the BEVs was ∼114 nm with a polydispersity index (PDI) of ∼0.237 (Figure 1(b): G). The particle charge measurement revealed a zeta‐potential of −23 mV, indicating the net negative surface charge of BEVs (Supporting Figure S-1).

Figure 1Structural and morphological characterization of BEVs secreted by *L. lactis*. (a) Schematic of workflow representing isolation and characterization of BEVs from *L. lactis* with or without antibiotic stress. (b) FESEM micrographs of *L. lactis* grown in GM17 broth showing budding out of BEVs from the bacterial surface. The early log phase of *L. lactis* (A) and the late stationary phase of *L. lactis* (B) showing the bud‐out event. FESEM (C), TEM (D), and cryo‐TEM (E) images of isolated BEVs showing a spherical morphology with a heterogeneous population. The scale bars are 200 nm (C, E) and 50 nm (D). Analysis of the mean diameter of BEVs, imaged by FESEM, TEM, and cryo‐TEM, showing the approximate size range from ∼10 to 100 nm (F). The DLS data show hydrodynamic diameter of BEVs with an average of ∼114 nm (G). Stability assessment of BEVs stored in phosphate‐buffered saline (PBS, pH 7.4) at room temperature (24°C; Mock), 30°C, and 40°C for 6 h, showing minor changes in size and particle concentration (H). Similarly, low‐temperature (−20°C) storage followed by two freeze–thaw cycles resulted in a minor increase in the particle size compared with the control (I). (c) Sterility assessment of BEVs isolated from *L. lactis* culture. GM17‐agar plate inoculated with BEVs showed no growth up to 48 h (A). To confirm the absence of endotoxin contaminants, freshly isolated BEVs were subjected to KDO (2‐keto‐3‐deoxyoctonate) quantification, which showed no detectable LPS/LOS in the test sample. A cell lysate of *C. jejuni* served as a positive control, while assay buffer (HEPES) was used as a negative control (B). The presence of a visible pellet in the test samples (medium inoculated with *L. lactis*), compared with the absence of a pellet in the mock controls (medium only), indicates the purity of the BEV sample (C). Analysis by NTA further affirmed the presence of nanosized vesicles in the test samples, whereas no such structures were visualized or quantified in the mock control (medium only) (D). (d) The SDS–PAGE image showing the protein profile of BEVs and the various cellular fractions (CWF, cell wall fraction; CF, cytoplasmic fraction; and WCL, whole‐cell lysate) of *L. lactis* (A). M: Molecular mass marker (KDa). Micro LC‐ESI‐MS/MS analysis of BEV proteins with different subcellular locations, and the pie chart presenting the percentage of proteins (B).

**Figure:**
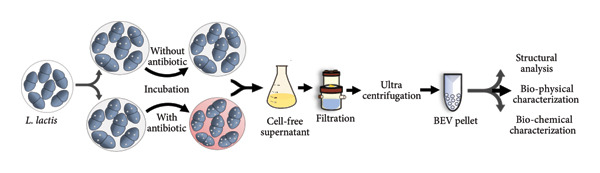
(a)

**Figure:**
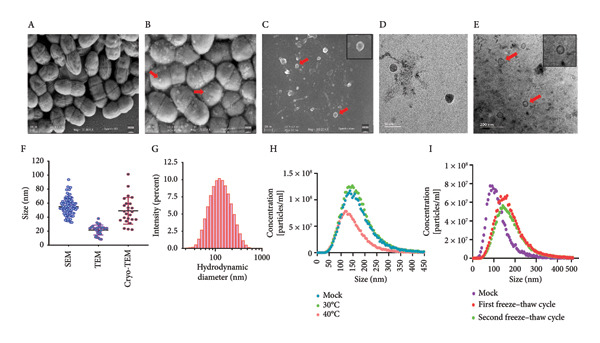
(b)

**Figure:**
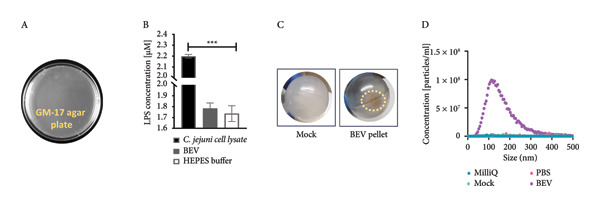
(c)

**Figure:**
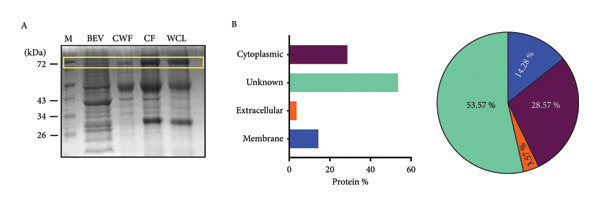
(d)

To further assess the effect of temperature on the storage stability of *L. lactis* BEVs, only minor variations in hydrodynamic diameter were observed, with no significant alterations in particle morphology under either low‐temperature conditions (−20°C) or elevated temperatures (30°C and 40°C for 6 h). These results confirm the stability of BEVs and their morphological consistency across different storage conditions (Figure 1(b): H‐I).

The SDS–PAGE analysis of BEVs showed that the proteins in the vesicles correspond to the various cellular fractions, which further affirmed that BEVs carry the proteins from the cell walls and protoplasm of the source bacteria (Figure 1(d): A). To further characterize the proteomic profile of BEVs, mass spectrometric analysis was performed using the UniProt database as a reference for *L. lactis*. A total of 28 proteins were identified in the BEV fraction, corresponding to the proteome of *L. lactis* subsp. *cremoris*. PSORTb analysis revealed that approximately 14% of these proteins originated from the membrane fraction, approximately 28% were cytoplasmic, approximately 53% had unknown localization, and approximately 3.5% were extracellular (Figure 1(d): B). Among the proteins with unknown localization were CHAP‐domain‐containing proteins, NHLM bacteriocin system ABC transporters, and C39 family peptidases, which are of note (Supporting Table S1). These findings suggest that BEV proteomes are enriched with significant contributions from cytoplasmic and membrane proteins of the source bacterium, as well as a smaller proportion of extracellular proteins.

The specific strain of *L. lactis* used in this study (*L. lactis* subsp. *cremoris* MG1363‐NZ9000) is generally considered endotoxin‐free. To confirm this, we evaluated the sterility of the isolated BEVs for both bacterial contamination and the presence of endotoxin (such as lypopolysaccharide [LPS] or lipooligosaccharide [LOS]). No bacterial growth was observed on agar plates inoculated with BEV samples (Figure 1(c): A, and no detectable levels of LPS were found by 2‐keto‐3‐deoxyoctonate (KDO) assay, confirming sterility and an endotoxin‐free status (Figure 1(d): B). Additionally, to rule out contamination from medium or washing buffers, mock controls (medium only) were processed in parallel with *L. lactis* samples. A visible pellet was observed only in the test samples, confirming that the pellets originated from bacterial cells and not from the medium or buffer components (Figure 1(d)).

### 2.2. Treatment of *L. lactis* With a Subinhibitory Dose of Ampicillin‐Enhanced BEV Biogenesis

The complexity and challenges associated with BEV biogenesis from Gram‐positive bacteria *L. lactis* make us curious to investigate the facilitation of BEV production. Previous studies have reported that microenvironmental stress could alter the BEV biogenesis process in bacteria. *L. lactis*–derived BEVs bearing lipid membrane and protein contents comparable to different cellular fractions of bacteria probably passed through the thick PGN layer before release. To investigate whether targeting PGN cross‐linking and the protein synthesis machinery could alter BEV biogenesis, this study selected two broad‐spectrum antibiotics based on their ability to modulate cell wall biosynthesis or the protein synthesis mechanism of bacteria. We cultured *L. lactis* in the presence of ampicillin (Amp) and chloramphenicol (Cm) at optimized concentrations of 0.1 and 1.5 μg/mL, respectively. These specific concentrations were chosen as sub‐MIC doses (MIC50 of ∼0.35 and 3.9 μg/mL, respectively) (Supporting Figure S-2). For both (antibiotic‐treated or untreated) conditions, BEVs were harvested from an equal volume of overnight‐grown culture medium (∼150 mL) by ultracentrifugation (120, 000 × *g*, 2 h) (Figure 1(a)). Following treatment with the indicated concentration of antibiotics, we observed a significant increase (approximately 3‐fold) in BEV numbers by NTA when *L. lactis* cells were treated with Amp (∼0.1 μg/mL) compared to untreated cells (Figure 2(a): A). In contrast, treatment with Cm (∼1.5 μg/mL) showed no significant change in BEV production (Figure 2(a): G). The comparative analysis of BEV contents suggests that the Amp treatment (0.1 μg/mL) of *L. lactis* cells substantially increased (approximately 5‐fold) the total protein content of BEVs, compared to Cm treatment (Figure 2(a): C, I). The size distribution of the isolated BEVs from both treatments remains consistent, showing no significant difference compared to the untreated (Figure 2(a): B, H). Similar to protein, BEVs isolated from Amp‐treated *L. lactis* cells showed a marked increase in the total lipid content (∼1.95‐fold), carbohydrate content (approximately 2‐fold), and peptyodoglycan content (∼3.5‐fold) (Figure 2(a): D–F) compared to Cm treatment (Figure 2(a): J–L) or untreated *L. lactis*. These findings suggest that Amp effectively influences BEV biogenesis compared to Cm treatment. Moreover, we confirmed that the optimal use of Amp preserves the viability of *L. lactis* cells as a source of BEVs, thereby supporting an efficient enrichment strategy. The TEM micrographs of Amp‐treated and untreated bacterial cells revealed the release of BEVs from the bacterial surface (Figure 2(c): A). To determine whether the morphology and the size distribution of BEVs were affected by the Amp treatment or not, BEV samples were subjected to DLS, SEM, and TEM analysis. The micrograph of isolated BEVs captured by FESEM (Supporting Figure S-3) and TEM revealed that BEVs from Amp‐treated *L. lactis* cells exhibit comparable shapes (spherical) with an average size of 80 nm (Figure 2(c): B). The hydrodynamic size distribution of Amp‐treated BEVs analyzed by DLS ranges from 30 to 300 nm (Figure 2(c): C). Furthermore, when checked for batch‐to‐batch variability, in terms of total protein content of BEVs (in either the presence or the absence of antibiotic treatment), the calculated coefficients of variation (CV) (13% without ampicillin treatment and 5% with ampicillin treatment) were found to be within the limit; hence, the observed variability is acceptable (Supporting Table: S-2).

Figure 2Effect of antibiotic stress on BEV biogenesis and comparative assessment of the biochemical compositions of BEVs. (a) Comparative analysis of BEV production from *L. lactis* after treatment with a sub‐MIC50 of antibiotics (ampicillin and chloramphenicol). The nanoparticle tracking analysis of isolated BEVs from antibiotic‐treated *L. lactis* showing a marked increase in the BEV concentration when treated with ampicillin (Amp) (A), while no noticeable change in the BEV concentration was observed when treated with chloramphenicol (Cm) (G). The analysis of the hydrodynamic diameter (size) of isolated BEVs (with or without antibiotic treatment) suggests no significant difference, irrespective of the antibiotic types (B, H). However, in the case of ampicillin treatment, the concentration of isolated BEVs was markedly increased as compared to the “no treatment” control. The compositional analysis of BEV by BCA assay (for protein), anthrone assay (for carbohydrate), sulfophosphor vanillin assay (for lipid), and muramic assay (for peptidoglycan) (Amp: C–F and Cm: I–L). Comparative analysis showing the Amp treatment of *L. lactis* (0.1 μg/mL) resulted in a significant increase in protein (C), carbohydrate (D), lipid (E), and peptidoglycan content (muramic acid) (F) of BEVs. In contrast, minor changes were observed in total protein (I), carbohydrate (J), and lipid content (K); however, a significant reduction in the peptidoglycan content (muramic acid) (L) was noted when treated with Cm (1.5 μg/mL). (b) The table represents the total amount (μg) of protein, carbohydrate, lipid, and peptidoglycan present in BEVs isolated from *L. lactis* treated with different antibiotics. Δ = Treated BEVs – Control BEVs. (c) TEM images of untreated and Amp‐treated *L. lactis* (scale bar=100 nm) (A). TEM micrograph of isolated BEVs by ultracentrifugation from Amp‐treated *L. lactis* culture (scale bar=200nm) (B). The size distribution of Amp‐treated BEVs, showing a mean hydrodynamic diameter of ∼approximately 164 nm (C).

**Figure:**
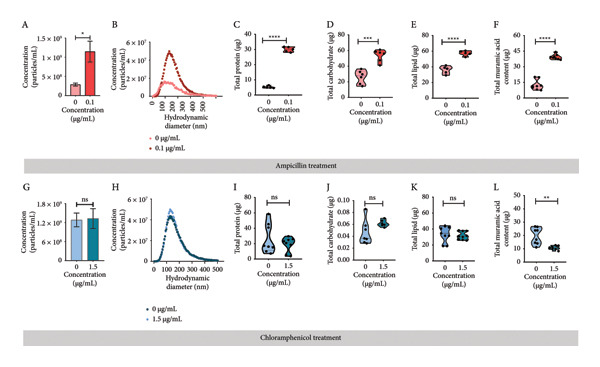
(a)

**Figure:**
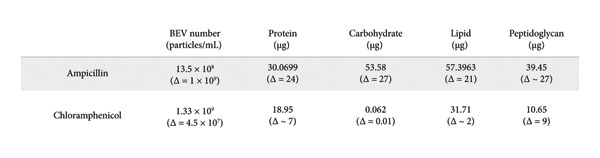
(b)

**Figure:**
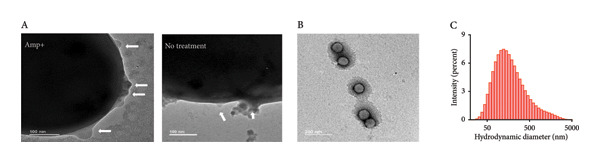
(c)

Taken together, present data indicate that antibiotic stress concerning protein synthesis has a minimal effect on BEV biogenesis, but the modulation of PGN cross‐linking promotes BEV production in *L. lactis*.

### 2.3. *L. lactis*–Derived BEVs Exhibit Efficient Internalization by Both Immune and Nonimmune Cells

#### 2.3.1. *In Vitro* Fusion With Artificial Vesicles (Liposomes)

Fusion is crucial for BEVs, as it enables the effective communication and delivery of cargo to target cells by bypassing degradation pathways. Membrane fusion or uptake of BEVs offers efficient targeting and bioavailability, improving their efficacy in therapeutic applications and immune modulation. Next, we assess the membrane fusion capability of *L. lactis*–derived BEVs using membrane‐mimetic artificial fluorophore‐tagged liposomes (DOPC/DOPE/DOPS/Chol/NBD‐PE/Rh‐PE) by FRET assay (DOPC: 1,2‐dioleoyl‐sn‐glycerol‐3‐phosphocholine; DOPE: 1,2‐dioleoyl‐sn‐glycero‐3‐phosphoethanolamine; DOPS: 1,2‐dioleoyl‐sn‐glycerol‐3‐phospho‐L‐Serine; Chol: cholesterol; NBD‐PE: 1,2‐dioleoyl‐sn‐glycero‐3‐phosphoethanolamine‐N‐[7‐nitro‐2‐1,3‐benzoxadiazol‐4‐yl); Rh‐PE: 1,2‐dioleoyl‐sn‐glycero‐3‐phosphoethanolamine‐N‐(lissamine rhodamine B sulfonyl]). A marked decrease in energy transfer and an increase in the donor fluorescence intensity indicate the efficient energy transfer from a donor fluorophore (NBD‐PE) to an acceptor fluorophore (Rh‐PE) at a physical proximity within 1–10 nm was hampered. The FRET analysis of incubated untagged BEVs with fluorophore‐tagged liposomes showed a modest increase in % fusion up to ∼12% within 30‐min incubation (Figure 3(a): A‐B). Next, the natural fusion propensity of the isolated BEVs was observed using TEM, followed by 48 h incubation at 4°C, in PBS buffer. The morphological changes, including the merged membrane bilayer and vesicle aggregation, resemble the inherent fusogenic property of these nanovesicles (Figure 3(a): C). Together, these data suggest that *L. lactis–*derived BEVs exhibit successful membrane integration and strong fusogenic attributes, facilitating their role in cytosolic cargo delivery.

Figure 3In vitro assessment of membrane fusion and cellular uptake of BEVs. (a) Assessing the membrane fusion ability of *L. lactis* BEVs. FRET assay was performed using fluorophore‐doped liposomes (DOPC/DOPE/DOPS/Chol/NBD‐PE/Rh‐PE in a 44/27/6/20/1.5/1.5 molar ratio) (A). Unprobed BEVs were mixed with the fluorophore‐doped liposomes at a ratio of 20:1, and fusion kinetics were measured by FRET dilution. The fusion efficiency of BEVs with probed liposomes increased to ∼22% over time, while the fusion between probed and unprobed liposomes was ∼11%. Probed liposomes only served as a control (B). The spontaneous fusion of BEVs in the physiological buffer was captured by TEM (C). (b) In vitro cellular uptake of Dil‐C18 (20 μg/mL)–labeled *L. lactis* BEVs by murine macrophage (J7774A.1) (A) and human adenocarcinoma cells (HeLa cells) (B) with the function of time. The merged images show a time‐dependent increase in the intracellular accumulation of BEVs. Each confocal micrograph represents BEVs (red fluorescence) and nuclei (blue fluorescence) stained with Dil‐C18 and DAPI, respectively. Scale bars are 20 μm. (c) Mechanism of the cellular uptake process. The mean fluorescence intensity (measured using Fiji software), showing early accumulation (∼3 h) of BEVs in J774A.1 cells compared to HeLa cells (∼6 h) (A). Clathrin and caveolin‐mediated (dynamin‐dependent) cellular uptake of BEVs was confirmed using dynasore, a chemical inhibitor for GTPase activity of dynamin (B). For both cell types (Macrophage J774A.1: top row; epithelial cells HeLa: bottom row), pretreatment with dynasore (80 μM) resulted in a marked reduction in fluorescence signals (green) (red: Phalloidin 647 staining cellular cytoskeleton; blue: DAPI staining nucleus; green: Dil‐C18–labeled BEVs) (scale bar=20 μM).

**Figure:**
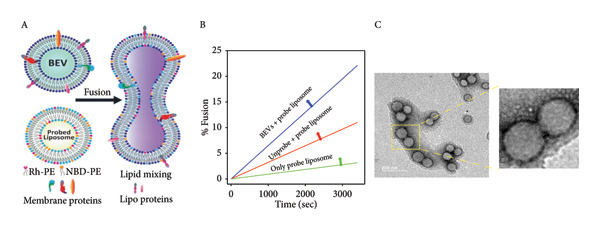
(a)

**Figure:**
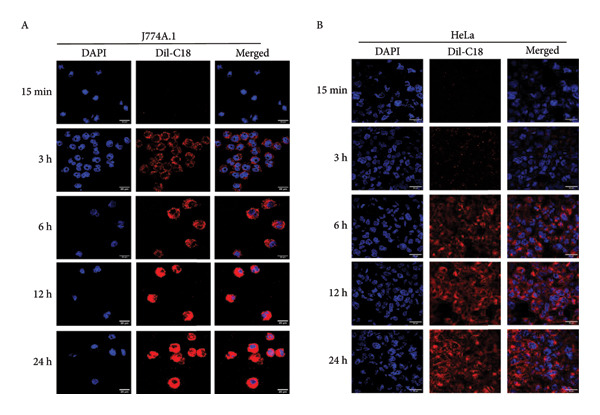
(b)

**Figure:**
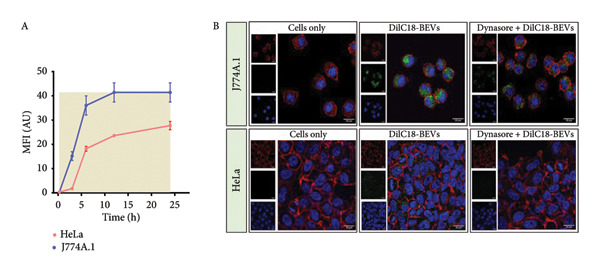
(c)

#### 2.3.2. Cellular Internalization

As a GRAS category probiotic bacterium, *L. lactis* (NZ9000) neither colonizes the gut nor invades host cells [13]. Given the importance of the intracellular uptake of BEVs intended for use as a drug carrier, we sought to investigate whether, as nonspherical vesicles, BEVs can be internalized by host cells. However, compared to artificial membranes, biological cell membranes are more dynamic; therefore, the uptake of nanosized vesicles through the cell membrane is more complex and regulated. Here, we choose immune (J774A.1) and nonimmune (HeLa) cells to study how BEVs interact with the target cells. Using lipophilic fluorescent dye 1,1’‐dioctadecyl‐3,3,3’,3’‐tetramethylindocarbocyanine perchlorate (dye Dil‐C_18_)–labeled BEVs, we demonstrated that BEVs can be efficiently internalized and accumulate within the cytoplasm of recipient cells over time (15 min–24 h postincubation) in both J774A.1 and HeLa cells (Figure 3(b): A‐B). The Dil‐C_18_ fluorescence intensity increased over time after the initial 3 h incubation, peaking at 12 h, with no significant increase observed at 24 h. This suggests that the cells continuously uptake the vesicles for up to 12 h, after which the uptake efficiency reaches saturation. The mean fluorescence intensity (MFI) of internalized BEVs analyzed by confocal laser scanning microscopy (CLSM) micrographs using Fiji software, taken at five different time points, indicates that J774A.1 cells exhibit early uptake of BEVs (as early as 3 h compared to 6 h for HeLa cells) than HeLa cells (Figure 3(c): A). Cellular uptake kinetics data indicate that BEV uptake occurs in a time‐dependent manner, with BEVs being highly effective in delivering cargo, maintaining their stability *in vitro*. This effective internalization of BEVs suggests their direct role in conveying biological information, imparting their effects to recipient cells, and strengthening their candidacy as delivery nanocarriers.

#### 2.3.3. Dynamin‐Dependent Endocytosis of *L. lactis–*Derived BEVs

Furthermore, we assume that the internalization of BEVs may utilize specific endocytic pathways to deliver the cargo to recipient cells. To elucidate the underlying mechanism of BEV uptake, we used chemical inhibitors to block specific endocytic trafficking pathways in both murine J774A.1 (immune) and human HeLa (nonimmune) cells. Using dynasore (a known inhibitor of clathrin‐ and caveolin‐mediated endocytosis [CME]), we observed a significant reduction in the intracellular localization of BEVs, as evidenced by CLSM images (Figure 3(c): B). Comparative analysis of fluorescence signals associated with Dil‐C_18_‐BEVs revealed that the reduction is significantly higher in the case of HeLa cells (∼53%) compared to J774A.1 cells (∼24%), suggesting that the uptake of BEVs was not majorly mediated by clathrin and caveolin‐dependent endocytosis in J774A.1 (Supporting Table: S3).

### 2.4. Evaluating the Therapeutic Efficacy of Drug‐Loaded BEVs

#### 2.4.1. The Antiproliferative Effect of Doxorubicin‐Loaded BEVs (BEV_DOX_) Is Comparable to That of the Free Drug

After checking the cargo delivery efficiency of BEVs, we were curious to check the potential of BEVs in drug carriage and delivery to the target site. To assess the efficacy of BEVs as a cancer drug carrier, we evaluated their biocompatibility using the MCF‐7 cell line. An MTT assay was performed after 48 h incubation with serial dilutions of BEVs, starting with 80 μg/mL, which showed no significant cell death (Supporting Figure S5A). To determine whether BEVs can effectively deliver the drug, we next encapsulated doxorubicin (a anthracycline class of drug) into BEVs using the electroporation method. The drug encapsulation efficiency was estimated to be ∼9%, and the fluorescence signal analysis by NTA showed ∼54% labeling of BEVs with doxorubicin (Supporting Figure S6G, S6C). NTA, FESEM, and TEM analyses of BEV_DOX_ revealed its stability and the absence of significant morphological changes (Supporting Figure S6D–S6F). To evaluate the antiproliferative efficiency of BEV_DOX,_ we performed a standard clonogenic assay using human MCF‐7 breast cancer cells with a subinhibitory dose of 10 g/mL DOX concentration (Supporting Figure S5B). The difference in cell survival ability was quantified by measuring the absorbance (*A*_590nm_) of poststained colonies formed from 7‐ to 8‐day incubation after 48 h of post‐treatment with free Dox or BEV_DOX._ Our data suggest approximately a 4‐fold and 3‐fold reduction in absorbance when the cells were treated with free Dox or BEV_DOX_ (10 ng/mL), respectively, compared to untreated cells (Figure 4(b): B‐C), highlighting that BEVs can efficiently deliver doxorubicin to tumor cells, thereby restricting cell proliferation.

Figure 4Efficacy of doxorubicin‐loaded BEVs (BEVDOX) against MCF‐7 cells. (a) Schematic showing experimental outline for drug loading and assessment of antiproliferative activity. (b) The drug encapsulation (Dox) efficiency of BEVs was calculated against a standard plot generated using different concentrations of free Dox (A). The purple line on the standard curve denotes the corresponding drug concentration used in further assays. Representative images of “colony forming assay” showing the viability of MCF‐7 cells treated with BEVDOX (10 ng/mL) or Dox alone (10 ng/mL) (B). Bar chart showing the MCF‐7 cell viability (MTT assay) based on the mean absorbance (A595)  ± SE of three biological replicates (^∗∗^ *p* ≤ 0.01), suggesting the comparable effect of BEVDOX or its free form (C). The release kinetics of the drug (Dox‐HCL)‐loaded BEVs were observed using the dialysis method in two different buffer conditions. One in physiological pH 7.4 and another in pH 5.5, which mimics malignant microenvironmental pH in the presence of 10 mM H_2_O_2_. The release of the drug into the buffer was measured by fluorescence emission (excitation: 475 nm, emission: 595 nm) of 50 μL of the buffer, at different time points, and then returned to the beaker after measurement (D). Flow cytometric analysis of MCF‐7 cell cycle arrest (CCA) following free Dox or BEVDOX treatment. Histogram showing cell accumulation in the sub‐G0/G1 (∼40%) phase when treated with free Dox, in contrast to G2/M‐phase (∼45%) arrest when treated with BEVDOX (E‐F). (c) Annexin‐V/PI apoptosis detection assay: To confirm apoptosis, drug‐treated cells were stained with FITC‐labeled Annexin‐V/PI and subjected to flow cytometric analysis (A–B). Data suggest marked induction of late (Q2: Annexin V FITC+/PI+) and early apoptosis (Q4: Annexin V FITC+/PI–) of the cells when treated with BEVDOX (0.6 μg/mL) or Dox (0.6 μg/mL) alone, in contrast to untreated cells. The bar plot represents the quantitative analysis of apoptosis (late + early), with data presented as the percentage of cell cycle distribution ± SE from three independent experiments. (^∗∗∗∗^ *p* ≤ 0.0001) (B). Effect of drug treatment on proliferation and migration of MCF‐7 cells (scratch assay) (C). Representative bright‐field microscopy images captured at different posttreatment time points (0, 6, 12, 24, and 48 h) showing a marked reduction in tumor cell proliferation and impaired cell migration by BEVDOX treatment. MCF‐7 cell viability by AO/PI double staining (D). Representative epifluorescence images of MCF‐7 cells (treated with BEVDOX or Dox alone, 0.6 μg/mL) stained with acridine orange (AO) and propidium iodide (PI), suggesting a comparable effect exhibited by BEVDOX or Dox alone. AO (502 nm and 525 nm) emits green fluorescence upon binding to the nucleus, and PI (535 nm and 615 nm) emits red fluorescence for dead cells and merged yellow‐denoted dead cells.

**Figure:**
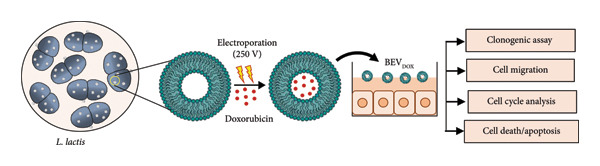
(a)

**Figure:**
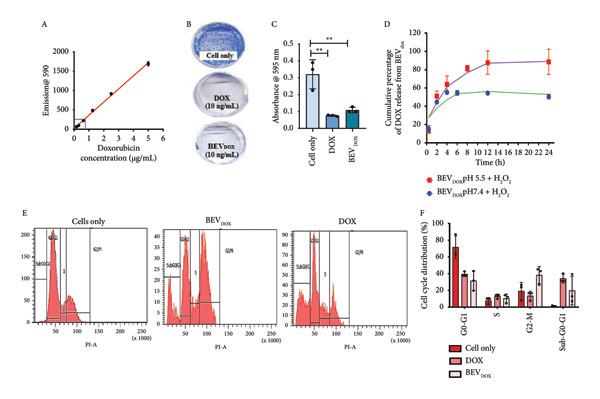
(b)

**Figure:**
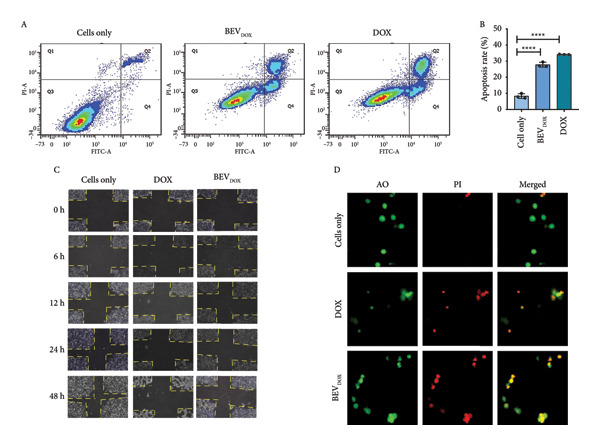
(c)

Next, we performed a 24‐h drug release kinetics study using doxorubicin‐loaded BEVs (BEV_DOX_) enclosed in a dialysis bag (cutoff 50 kDa) under two PBS conditions: pH 7.4 and pH 5.5. The results showed nearly complete drug release at pH 5.5, whereas release was comparatively slower at pH 7.4 (Figure 4(b): D).

Since doxorubicin inhibits cell proliferation primarily through cell cycle arrest at the G2/M phase and triggers apoptosis, we further performed the flow cytometric analysis of the treated cells at 36 h post‐treatment. Applying Dean–Jett–Fox univariate cell cycle analysis by flow cytometry, we demonstrated that BEV_DOX_ triggers the growth inhibition of MCF‐7 cells primarily through the induction of G(2)‐/M‐phase cell cycle arrest (∼40%) and accumulation of cells in sub‐G0/G1 (∼20%)‐phase compared to untreated cells (Figure 4(b): E‐F). Additionally, an Annexin‐V FITC apoptosis assay was performed to assess apoptosis by flow cytometric analysis. A significant increase in fluorescence signals in both the early (Q4) and late apoptosis (Q2), with cumulative apoptosis rates of ∼27% for BEV_DOX_‐treated cells, while ∼34% in the case of free Dox treatment was observed (Figure 4(c): A‐B). Next, to examine the effect of BEV_DOX_ on cell migration and proliferation, we performed a standard scratch assay using a confluent monolayer of human MCF‐7. The pattern of cellular migration toward the “cell‐free area” was found to be impaired and remained consistent throughout the experimental period (0–48 h post‐treatment) (Figure 4(c): C). To determine the impact of treating MCF‐7 cells with BEV_DOX_, we employed the propidium iodide (PI) and acridine orange (AO) double staining method. The epifluorescence images confirmed enhanced PI signals (in red) in the BEV_DOX_‐treated cells, indicating efficient DNA damage by the drug, which led to significant cell death (Figure 4(c): D). Together, our data suggest that BEV_DOX_ can efficiently control tumor cell proliferation, restrict tumor cell migration, and drive apoptosis, confirming BEVs as effective drug delivery carriers.

#### 2.4.2. Gentamicin‐Loaded BEVs (BEV_GEN_) Effectively Inhibit the Growth of Methicillin‐resistant Staphylococcus aureus (MRSA) and *C. jejuni*

Based on their nanosized vesicular structure and ability to undergo both cytosolic and endosomal pathways for cellular uptake, we hypothesize that *L. lact*is BEVs could serve as a promising platform for drug delivery against both extracellular and intracellular pathogens. For this, as a model drug, we encapsulated gentamicin into the BEVs by electroporation and tested its (BEV_GEN_) efficacy against MRSA (a Gram‐positive extracellular bacterium) and *C. jejuni* TGH9011 (a Gram‐negative intracellular bacteria). The drug loading capacity of BEVs, as revealed by the ninhydrin assay (ninhydrin reacts with the free amine groups of the gentamicin to form a ninhydrin–gentamicin complex and can be measured at *A*_418_), was found to be ∼25% (Figure 5(a): B, C). Further analysis of drug‐loaded BEVs by NTA revealed that the mean hydrodynamic size of BEV_GEN_ was ∼125 nm, and TEM micrographs showed no noticeable difference in the morphology, suggesting stability and similar morphological attributes to the untreated BEVs (Figure 5(a): D‐E).

Figure 5Antimicrobial effect of gentamicin‐loaded BEVs (BEV_GEN_) against MRSA and *C. jejuni.* (a) Schematic showing experimental outline for drug loading and assessment of antibacterial activity against MRSA and *C. jejuni* (A). To determine the drug encapsulation efficiency, a standard plot was generated for gentamicin using known concentrations of the drug by the standard ninhydrin test. The amount of drug present in BEV_GEN_ was calculated by plotting the absorbance value against standards (B), while the bar represents the encapsulation efficiency of gentamicin in BEVs (C). NTA represents the size distribution of BEV_GEN_ after electroporation (∼123 nm) (D). Representative micrographs of BEV_GEN_ captured by TEM (E). (b) Effect of BEV_GEN_ treatment on MRSA and *C. jejuni* growth: To evaluate the antibacterial activity of BEV_GEN_, a serial dilution of BEV_GEN_ started from 1 mg/mL (for MRSA) and 100 μg/mL (for *C. jejuni*) was tested in the solid phase (agar well diffusion assay) and for the liquid phase (microbroth broth dilution) starting from 12 μg/mL. Representative agar plate images showing the zone of inhibition of bacterial lawn treated with free drug or BEV_GEN_ against MRSA (A) and *C. jejuni* (D). Determining MIC50 of gentamicin and BEV_GEN_ against MRSA and *C. jejuni* (TGH9011) by liquid‐phase assay by the microbroth dilution method. After 24 h of drug treatment, the absorbance of the bacterial culture medium was measured at 600 nm (A600). The percentage of growth was calculated relative to untreated bacterial cultures (control). For MRSA, the MIC50 of the free drug and BEV_GEN_ were ∼approximately 0.04 and 0.01 μg/mL, respectively (B). For *C. jejuni* (TGH9011), the MIC50 of free drug and BEV_GEN_ were ∼0.8 and ∼0.09 μg/mL, respectively (E). Representative FESEM micrographs of MRSA (C) and *C. jejuni* (F) showing deflated/ruptured bacterial cells (yellow arrow) after treatment with a free drug or BEV_GEN_. The inset showing the deformed membrane of bacterial cells. Scale bar: 1 μm.

**Figure:**
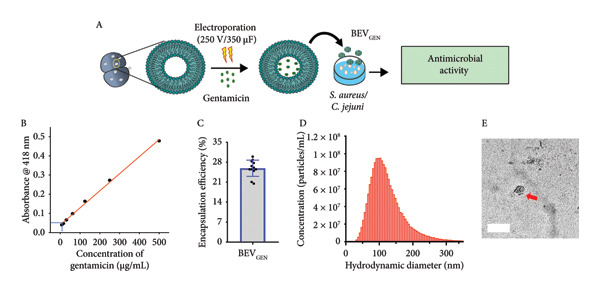
(a)

**Figure:**
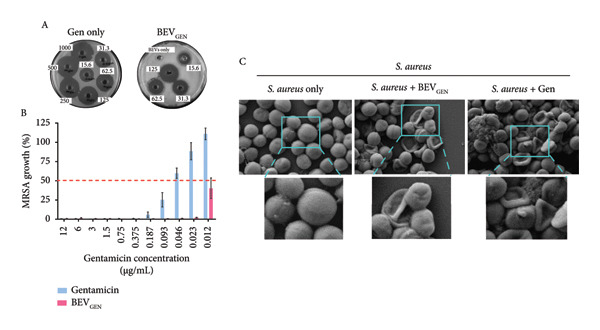

**Figure:**
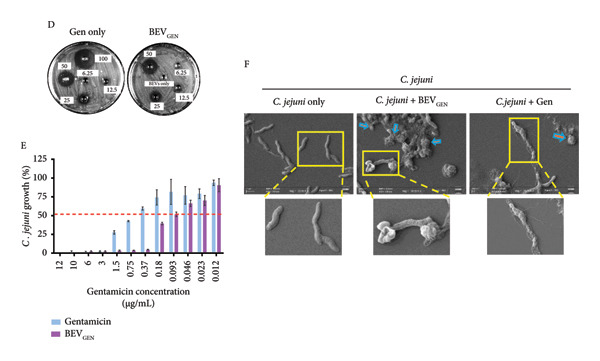
(b)

In terms of antibacterial activity against MRSA, we performed both solid‐phase and liquid‐phase analyses using a range of gentamicin concentrations present in BEV_GEN_ (for the solid phase: drug concentration range, ∼15.6–125 μg/mL; for the liquid phase: ∼12 μg to 12 ng/mL). In both cases, we observed a concentration‐dependent inhibition of MRSA growth following treatment with BEV_GEN_ (Figure 5(b): A‐B, Supporting Figure S-7A). Based on the microdilution method (liquid phases), MIC_50_ was calculated to be ∼0.04 μg/mL (free gentamicin) and ∼0.01 μg/mL for BEV_GEN_.

Similarly, we confirmed the concentration‐dependent antibacterial activity of BEV_GEN_ against *C. jejuni* in both solid and liquid phases (Figure 5(b): D‐E, Supporting Figure S7B). However, MIC_50_ (determined using the liquid‐phase microdilution method) was found to be ∼0.8 μg/mL (free gentamicin) and ∼0.09 μg/mL for BEV_GEN_.

The observed effects of BEV_GEN_ treatment on MRSA and *C. jejuni* were further validated through FESEM micrographs captured at the post‐treatment time point. The images revealed significant membrane deformation and structural damage in both bacterial species. In MRSA, severe membrane disruption was evident, characterized by severely damaged cellular architecture (zoomed view, Figure 5(b): C). Likewise, in *C. jejuni*, BEV_GEN_ treatment resulted in significant membrane damage and a transition to a coccoid form (orange arrow, Figure 5(b): F), indicating stress. These findings suggest that BEVs can effectively interact with and deliver drugs to both Gram‐positive and Gram‐negative bacteria.

#### 2.4.3. Amphotericin B–Loaded BEVs (BEV_AmB_) Demonstrate Significant Antifungal Activity Against *C. albicans*

To test whether the function of BEVs can be extended against delivery antifungals (such as Amphotericin B: AmB), we chose to use *C. albicans* as a dimorphic opportunistic fungal pathogen for interacting with and facilitating cargo delivery in fungal cells. Here, AmpB was loaded into BEVs (BEV_AmB_) by sonication, followed by G‐50 column chromatography to remove the free drug (Figure 6(a)). To determine the loading capacity, absorbance at 328 nm was measured, and the AmB encapsulation efficiency of BEVs was found to be ∼19% (plotted against the AmB standard curve) (Figure 6(b): A‐B). Further NTA of BEV_AmB_ suggests a mean hydrodynamic diameter of 113 nm (Figure 6(b): C), while TEM confirmed the consistency of BEV_AmB_ morphology compared with empty BEVs (Figure 6(b): D). To evaluate the antifungal efficacy of BEV_AmB_, an agar well diffusion assay was performed using varying concentrations of AmB encapsulated within BEVs (ranging from 12.5 to 50 μg/mL) and compared with free AmB (3.15–100 μg/mL) (Figure 6(c): A‐B; Supporting Figure S7C). Representative plate images demonstrated a concentration‐dependent increase in the zone of inhibition against *C. albicans*. This observation was further supported by morphological alterations, including a reduced number of spherical cells and the presence of multiple scars at the cell poles (red arrows), indentations on oval blastoconidial mother cells (yellow arrows), and deformed budding structures (green arrows) (Figure 6(c): C).

Figure 6Antifungal activity of Amphotericin B–loaded BEVs (BEV_AmB_) against *C. albicans*. (a) Schematic showing experimental outline for drug loading, purification, and assessment of antifungal activity against *C. albicans*. (b) The drug encapsulation (AmB) efficiency of BEVs was calculated against a standard plot generated using different concentrations of free AmB based on the absorbance (A). The bar plot represents the encapsulation efficiency of AmB in BEVs packaged by sonication (B). The size distribution of BEV_AmB_ was measured by NTA (∼119 nm) (C), and the morphology of purified BEV_AmB_ was captured by TEM (D). (c) Antifungal effect of BEV_AmB_ on *C. albicans* by solid‐phase assay (agar well diffusion assay) showing a clear zone of inhibition of fungal lawn culture by AmB alone or BEV_AmB_ using a 2‐fold serial dilution (A, B). The FESEM micrographs of free AmB or BEV_AmB_‐treated *C. albicans* showing multiple polar blemishes (indicated by red arrow) and deformed cells with indentations. Insets showing indentations (yellow arrow) and deformed buddings (green arrow) on oval blastoconidial mother cells after treatment with the AmB alone or BEV_AmB_ (C) (scale bar: 2 μm).

**Figure:**
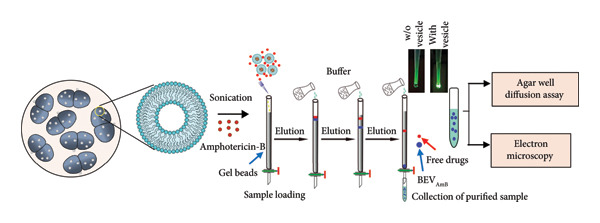
(a)

**Figure:**
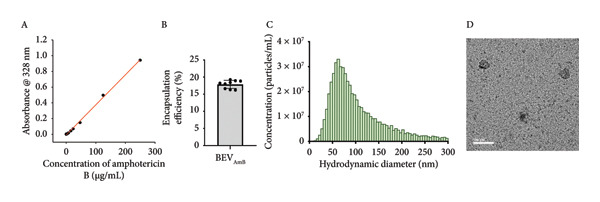
(b)

**Figure:**
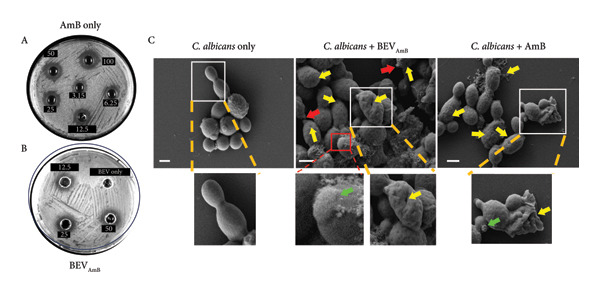
(c)

## 3. Discussion

The field of drug delivery is undergoing a rapid, transformative shift, with significant advancements aimed at enhancing therapeutic efficacy while minimizing off‐target effects. One of the most promising developments in this domain is the emergence of bioinspired drug delivery systems, particularly EVs, which include OMVs from Gram‐negative bacteria and exosomes (EXO) from eukaryotic cells [14–17]. These vesicles play crucial roles in various biological processes, such as cell communication, cargo transport, and immune modulation, making them attractive candidates for advanced drug delivery applications [18]. Historically, OMVs derived from Gram‐negative bacteria have been extensively studied due to their distinct surface architecture, which favors easy vesicle biogenesis through membrane blebbing [19]. In contrast, EVs from Gram‐positive bacteria (commonly known as BEVs) have remained understudied due to the intrinsic challenges associated with their thick cell wall composition, which makes their isolation and purification difficult. Generally, Gram‐negative bacteria release OMVs efficiently through the “pinching off” of the outer membrane [20].

In contrast, due to the thick PGN layer in Gram‐positive bacteria, the biogenesis of BEVs is more complex [21]. However, recent studies have confirmed that Gram‐positive bacteria, including *Staphylococcus aureus, Bacillus subtilis*, and *Lactobacillus* species, can produce OMVs in the form of nanosized vesicles (10–120 nm). It has been reported that inhibiting the transpeptidase enzymes of Gram‐positive bacteria can disrupt the cross‐linking of PGN layers [22–26]. This disruption can weaken the cell wall, cause the arrest of cell division, or increase turgor pressure, all of which may facilitate BEV biogenesis [9, 10]. However, most prior studies have utilized pathogenic bacteria as the source of BEVs, posing risks due to potential contamination with toxic subunits or metabolites from the parent strain [10].

We hypothesize that given the compositional similarities among various Gram‐positive bacteria, BEVs from probiotic bacteria could be promising, as they are generally endotoxin‐free and can directly interact with mucosal surfaces, a critical site for host–microbe interactions. In the present study, we envisioned using food‐grade probiotic bacteria *L. lactis* as a source for BEVs to showcase their potential as a drug delivery vehicle. A key advantage of using *L. lactis–*derived BEVs is the absence of LPS or LOS, as well as other toxic metabolites that are typically present in EVs derived from pathogenic Gram‐negative bacteria, which are responsible for endotoxin effects and often pose significant regulatory challenges. However, although not directly tested, the possibility of EVs carrying over DNA or RNA fragments from the source bacteria cannot be ruled out [27–33].

Considering the existing challenges of BEV isolation from Gram‐positive bacteria, in the first part of this study, we optimized an enrichment protocol for BEVs released from *L. lactis* using a subinhibitory dose of the β‐lactam antibiotic ampicillin. We demonstrated that targeting PGN biosynthesis with ampicillin can increase PGN carryover in BEVs, suggesting that ampicillin‐mediated inhibition of the PGN‐cleaving enzyme/s leads to enhanced BEV production of Gram‐positive bacteria. Consistent with our findings, previous research has shown that prophage‐encoded holin–lysin can also enhance BEV production in *L. lactis* [34]. Compared to amphenicol antibiotics like chloramphenicol, which inhibit protein synthesis by binding to the 50S ribosomal subunit, we show that ampicillin treatment can trigger a significant increase in the number and compositional abundance of proteins, lipids, carbohydrates, and muramic acid content in BEVs. This suggests that ampicillin may be a more rational choice for BEV enrichment over amphenicol antibiotics, as it enhances the BEV yield without adversely affecting the growth or survival of the source bacteria. This observation further supports the influence of antibiotics as microenvironmental stressors on bacterial physiology and the subsequent yield of BEVs, suggesting that physiological stress can regulate the production and compositional diversity of BEVs [35]. Moreover, we also report that *L. lactis*–derived BEVs maintain stability under both cold and elevated temperature conditions, supporting their potential for reliable handling, storage, and industrial applicability.

Since efficient uptake is crucial for effective intracellular drug delivery, we next attempted to understand the biological pathways involved in the cellular internalization of BEVs. We hypothesized that, as lipid‐bound vesicles enriched with a range of bacterial surface‐expressed, cytosolic, and secreted proteins, *L. lactis–*derived BEVs could play a more direct role in cellular communication [36]. This has led us to further venture into the vesicular‐cellular interaction process to understand the functional attributes of BEVs. Using the FRET analysis, we first examined their fusion ability with artificial membrane vesicles, such as liposomes. The membrane fusion kinetics of BEVs with liposomes suggest ∼22% fusion within 1.0 h, indicating that BEVs may be fusogenic, similar to other types of bacterial vesicles [37]. Furthermore, TEM micrographs of isolated BEVs incubated in PBS revealed membrane merging (self‐coalescence), suggesting their inherent fusogenic properties. However, further investigation is required to determine whether these characteristics enhance their credibility as drug delivery platforms. Nevertheless, without any external agitation or fusion‐inducing agent, this behavior suggests that BEVs can undergo spontaneous fusion, which may contribute to inter‐ and intraspecies BEV interaction/communication.

Compared to artificial membranes (such as liposomes), biological cell membranes are inherently dynamic, making the uptake of nanosized vesicles dependent on factors such as size, shape, surface charge, and target cell type. Therefore, in the next step, we attempted to explore the membrane fusion ability of BEVs using two different cell types: murine macrophage cells (J774A.1) and human adenocarcinoma (HeLa) cells. CLSM microscopy of cells incubated with DiL‐C_18_–labeled BEV showed a time‐dependent, steady, and consistent increase in the intracellular accumulation of BEVs for both cell types. These findings underscore that, despite *L. lactis* being noncolonizing and noninvasive, BEVs released from them exhibit the capacity to interact and fuse with diverse host cell types, thereby enabling efficient cytosolic delivery of their molecular cargo. This observation is consistent with our previous studies, where we reported the efficient cellular uptake of EVs from Gram‐negative bacteria [16]. However, several other studies reported that BEV uptake is often cell‐specific. For example, the treatment of HT29 cells with chlorpromazine and dynasore markedly blocks the cellular entry of *L. plantarum* BGAN8 BEVs, highlighting the prominent role of CME in the uptake of BEVs [38]. However, unlike previous studies, which have primarily focused on nonimmune cells, our findings demonstrate that *L. lactis* BEVs utilize similar uptake pathways in both immune (J774A.1) and nonimmune cells (HeLa). Notably, in immune cells, CME‐mediated uptake was relatively lower (24% vs. 53%), suggesting the involvement of phagocytosis or additional endocytosis pathways.

Together with their intriguing morphological, physiological, and functional attributes, *L. lactis* BEVs hold significant promise as a novel drug delivery platform, facilitating the transport of diverse bioactive molecules and therapeutic cargo to target cells. Since LAB vectors secrete several antimicrobials, such as bacteriocins, nisin, and lactococcin, these can naturally incorporate into BEVs, further enhancing the targeted antimicrobial activity and maximizing the therapeutic benefits of specific candidate drugs [39]. Therefore, we propose that if BEVs retain the key probiotic properties of their source bacteria, such as natural adjuvanticity, minimal immunogenicity, and broad health‐promoting benefits, they hold strong potential as promising delivery vehicles for a wide range of therapeutic agents [40].

Based on these anticipations, we hypothesized that BEVs could serve as a multimodal drug delivery platform capable of targeting both cancer cells and microbial pathogens. To achieve this, we established an optimal drug encapsulation strategy, determined encapsulation efficiency, and assessed the *in vitro* effects of drug‐loaded BEVs on cancer cell proliferation and microbial growth (*S. aureus, C. jejuni*, and *C. albicans*). The exogenous loading of doxorubicin, gentamicin, and Amphotericin B was successfully achieved with an encapsulation efficiency of ∼9%, 25%, and 19%, respectively, enabling us to evaluate their in vitro therapeutic efficacy. We demonstrate that doxorubicin‐loaded BEVs (BEV_DOX_) exhibit considerable efficacy (compared to the free drug) in terms of cell cycle arrest, leading to effective progression into the sub‐G0/G1 phase, which suggests the effective activation of apoptotic pathways. We further confirmed that BEV_DOX_ treatment (a subinhibitory dose of 0.6 μg/mL) of human MCF‐7 cells can increase the number of Annexin‐V/PI double‐positive cells (∼16%), indicating late apoptosis of the treated cells (Figure 4(c): B). Additionally, ∼12% of cells were found to be Annexin‐V positive only, suggesting early apoptosis characterized by phosphatidylserine (PS) externalization and changes in membrane composition. In contrast, free Dox treatment (0.6 μg/mL) triggered ∼11% early apoptosis and ∼22% late apoptosis. The lower percentage of late apoptosis observed with BEV_DOX_ treatment suggests the possibility of prolonged drug retention by BEVs and delayed progression of apoptosis.

To explore the potential of *L. lactis* BEVs for antimicrobial delivery, we encapsulated gentamicin (as an antibacterial agent) and Amphotericin B (as an antifungal agent) and evaluated their efficacy against MRSA, *C. jejuni*, and *C. albicans*, respectively. We chose to use gentamicin for MRSA and *C. jejuni*, as it is a known aminoglycoside commonly used in clinical settings due to its potent bactericidal effects. However, frequent aminoglycoside use is associated with nephrotoxicity, leading to complications such as tubular necrosis, fibrosis, glomerular congestion, perivascular edema, and inflammation. To mitigate these adverse effects, targeted drug delivery systems are essential for optimizing dosing and minimizing toxicity [41]. This study investigated whether gentamicin‐loaded BEVs (BEV_GEN_) could effectively eliminate MRSA and *C. jejuni* at lower therapeutic doses. Our *in vitro* experiments demonstrated that BEV_GEN_ achieved antibacterial effects comparable to free gentamicin. These findings suggest that BEVs can efficiently deliver antibiotics and induce significant bacterial membrane disruption in both extracellular and intracellular pathogens. Since biofilm formation by MRSA and *C. jejuni* presents an additional challenge for conventional drug delivery methods, further investigation is warranted to determine whether gentamicin‐loaded BEVs can disrupt biofilms and access these hard‐to‐treat pathogens.

On the other hand, we used Amphotericin B (AmB), a well‐known antifungal antibiotic widely used to treat fungal and yeast infections, including those caused by *C. albicans* and *Cryptococcus neoformans* [42]. However, its clinical use in free form is often limited by severe side effects such as hypotension, delirium, fever, nausea, vomiting, abdominal pain, anorexia, headache, and thrombophlebitis. Additionally, extensive use of AmB is associated with side effects of hematological complications, including hypochromic normocytic anemia, leukopenia, and thrombocytopenia [43]. To test the potential of BEVs as a delivery vehicle for AmB, we demonstrated that BEV_AmB_ is capable of effectively disrupting the integrity of fungal cell walls, comparable to the free drug, against *C. albicans*.

Together, our findings demonstrate that drug‐loaded BEVs isolated from food‐grade probiotic bacteria can exhibit significant therapeutic benefits against cancer cell proliferation and multidrug‐resistant microbial pathogens. However, while the present study establishes the physicochemical characterization, purity, and *in vitro* performance of optimally produced BEVs from *L. lactis* cells, *in vivo* validation is essential to confirm their translational potential. Future studies will focus on evaluating BEVs in relevant infection or disease models to systematically investigate *in vivo* safety, biodistribution, clearance, and therapeutic efficacy. Such investigations will be critical to substantiate the advantages of BEVs as a novel drug delivery platform and to bridge the gap between preclinical findings and clinical translation. Nevertheless, our findings demonstrate that optimizing *L. lactis* BEV enrichment and utilizing it as a drug delivery vehicle presents a promising alternative to conventional carriers, positioning it as a valuable natural nanocarrier for clinical and biomedical applications.

### 3.1. Conclusion

While artificial nanocarriers have demonstrated safety and efficacy, challenges such as high synthesis costs, limited shelf life, *in vivo* instability, and uncertain biological fate persist. In contrast, probiotic‐derived BEVs offer superior biocompatibility and safety. The current methods for isolating BEVs from Gram‐positive bacteria are technically demanding and often yield low quantities. Our study presents an optimized, cost‐effective protocol for BEV enrichment, suitable for both small‐scale research and scalable industrial applications. To achieve improved therapeutic outcomes, further modifications are needed to develop BEVs as targeted delivery systems with minimal off‐target effects or collateral damage. Future studies should explore strategies such as bioengineering BEVs or surface modifications via synthetic conjugate chemistry to create hybrid platforms, leveraging the strengths of BEV‐based drug delivery for optimal targeted therapy.

## 4. Materials and Methods

### 4.1. Bacterial Strain and Culture Conditions

*L. lactis* subsp. *cremoris* MG1363 (NZ9000) was a kind gift from Dr. Luis G Bermúdez Humar’an, French National Institute for Agricultural Research, Paris, France. *L. lactis* was grown in the M17 medium (HiMedia, India) supplemented with 0.5% glucose (w/v) (GM‐17 medium) at 30°C under static conditions. The Minimal Inhibitory Concentration (MIC_50_) of ampicillin and chloramphenicol against *L. lactis* was determined by the broth dilution method in 96‐well plates (Supporting Figure S2). The optimal sub‐MIC of antibiotic used for inducing BEVs biogenesis by *L. lactis* was fixed at 0.1 μg/mL (for ampicillin) and 1.5 μg/mL (for chloramphenicol) and added at the early log phase.

For the harvest of *C. jejuni* TGH 9011(NR‐4082, BEI Resources), Mueller–Hinton broth was used (MH broth, HiMedia) at 37°C under 10% CO_2_, 5% O_2_, and 85% N_2_. MRSA, a kind gift from Dr. Samiran Bandyopadhyay, IVRI Eastern Regional Station, Belgachia, Kolkata, and *C. albicans* CAI4‐F2‐Neut5L‐NAT1‐mCherry‐GFP (NR‐51634, BEI Resources) were grown in LB broth (HiMedia) and M‐BCG yeast and mold broth (HiMedia), respectively, at 37°C, under shaking conditions.

### 4.2. Cell Lines and Culture Medium

HeLa cell was procured from the National Centre for Cell Science (NCCS, Pune, India, RRID: CVCL_0030), MCF‐7 (RRID: CVCL_0031), and J774A.1 (RRID: CVCL_0358) were provided by Dr. Arnab Gupta (Department of Biological Sciences, IISER Kolkata). All cells were tested against mycoplasma and maintained in Dulbecco’s modified Eagle’s medium (DMEM; Gibco) with 10% (v/v) fetal bovine serum (Gibco) and 1% penicillin and streptomycin (P/S) supplementation (Gibco) at 37°C, 5% CO_2_.

### 4.3. Isolation of BEVs From *L. lactis*

The BEVs were isolated from *L. lactis* grown in a 150‐mL culture medium in the presence or absence of ampicillin (0.1 μg/mL) or chloramphenicol (1.5 μg/mL) treatment after reaching an optical density (OD_600 nm_) of ∼0.3 and were incubated overnight at 30°C. Next, the cell‐free culture supernatant was harvested by centrifugation at 8000 × *g* for 30 min at 4°C, followed by filtration through a 0.22‐μm vacuum filter (Cole‐Parmer, USA). Next, the filtered supernatant was ultracentrifuged at 120, 000 × *g* for 2.5 h at 4°C using a SW28 rotor (Beckman Instruments, USA) to pellet the BEVs. The pellet was finally resuspended in 1X PBS after washing with PBS using a SW‐41 Ti rotor at 200, 000 × *g* for 2 h at 4°C. For large‐scale BEV cultivation, 1 L of ampicillin‐treated cultures was grown, centrifuged, filtered, and concentrated using a 100‐kDa pressure‐based sample concentrator (Amicon Stirred Cells, USA) and further processed according to the abovementioned procedure. All experiments were conducted within one week after BEV isolation.

### 4.4. Hydrodynamic Diameter and Zeta (*ζ*) Potential of BEVs

To analyze the size distribution (hydrodynamic diameter) and net surface charge of BEVs, DLS was measured using a Nanosizer NanoZS Instrument (Malvern, USA) as described earlier [17]. For determining the size distribution and zeta‐potential, BEVs were diluted in 1 mL of MilliQ water at 1:100 and 1:20 ratios, respectively. The sample was further sonicated in a water bath sonicator (Branson, Fisherbrand, 50/60 Hz) and then filtered using a 0.22‐μm membrane filter. The data were collected as the average of three technical replicates, each with ten scans, with a 0.237 PDI value (for size) and a water refractive index as parameters.

### 4.5. Morphological Analysis of *L. lactis*–Derived BEVs

#### 4.5.1. TEM and Cryo‐TEM

The morphology of BEVs was examined by TEM and cryo‐TEM using a JEOL JEM‐2100 plus series electron microscope (Japan) as described previously [17]. Briefly, 10 μL of isolated BEVs were cast on a carbon formvar‐coated mesh copper grid and was allowed to absorb for 15 min at RT. The sample was stained with 1.5% uranyl acetate for 2 min and then rinsed twice with MilliQ water, followed by air and vacuum drying. The sample was directly observed under the electron microscope at 120 KV. For cryo‐TEM sample preparation, 5‐μL BEVs were cast on the copper mesh grid held on Cryoplunge (Gatan), followed by negative staining, and subjected to simultaneous plunging into a precooled liquid ethane reservoir (−180°C). Next, the fixed sample was transferred to a cryoholder and imaged using a CCD camera on a JEOL JEM‐2100 Plus, maintaining a temperature of −180°C.

#### 4.5.2. FESEM

To visualize the budding process of BEVs from bacterial surfaces, FESEM micrographs of bacterial samples were captured using a previously published protocol with some minor modifications [44]. Briefly, an overnight‐grown *L. lactis* culture was centrifuged at 2000 × *g* for 5 min, followed by a PBS wash. The sample was fixed with 2.5% glutaraldehyde (SRL, India) for 1 h, followed by resuspension in fresh PBS. Then, a drop was cast onto a sterile coverslip and air‐dried. Next, the sample was dehydrated using a series of graded ethanol solutions (35%, 50%, 70%, 90%, and 100%) for 10 min at each step. After drying, the sample was vacuum‐dried overnight, sputter‐coated with platinum and imaged using a Carl Zeiss SUPRA 55 FESEM.

To visualize the structure of BEVs, the isolated BEVs were diluted to a 1:10 ratio in PBS, sonicated (using a water bath sonicator) for 15 min, and drop‐cast onto a glass coverslip. After vacuum drying overnight, the sample was coated with platinum using a sputter coater. The images were acquired using a Carl Zeiss SUPRA 55 FESEM.

#### 4.5.3. Quantification and Size Distribution of BEVs

The concentrations of freshly isolated BEV were measured using a nanoparticle tracking analyzer (NTA) in NanoSight Pro (Malvern PANalytical, USA), following published methods with some modifications [45]. In brief, the isolated BEV sample was prepared in 1.0 mL of MilliQ water, thoroughly sonicated for 10 min in a bath sonicator (50/60 Hz), and then passed through a 0.22‐μm syringe filter. The sample was then loaded onto the NanoSight syringe, and the required settings were established according to the provided software (NanoSight NS300). For each measurement, ten 60‐s video captures were taken using light scattering at 532‐nm laser scanning, with a syringe speed of 3 μL/min maintained. The size and concentration of the BEVs were analyzed using the in‐built NanoSight software NS XPLORER, v1.1.0.6.

#### 4.5.4. Assessing the Sterility of BEVs Isolated From *L. lactis* Culture

To evaluate the sterility of BEVs, freshly harvested samples collected from the pellet formed in the ultracentrifuge tube were washed, reconstituted in PBS (pH 7.4), and plated onto GM17‐agar plates. The plates were then incubated for 48 h at 30°C or 37°C to detect any bacterial/fungal growth.

Next, to determine whether the observed pellets in the ultracentrifuged tube originated from bacterial cells rather than from the media components, we processed the mock medium (medium only) alongside the test samples (medium inoculated with *L. lactis*) and checked either for a visible pellet at the bottom of the centrifuge tube or subjected to NTA.

To evaluate endotoxin contamination, if any, freshly isolated BEVs were subjected to KDO quantification, following a standard protocol [46] with minor modifications. Briefly, BEV samples were hydrolyzed with 0.04 M sulfuric acid at 100°C for 25 min to cleave the glycosidic linkages and release KDO from the LPS molecules. After cooling to room temperature, the hydrolysates were treated sequentially with 0.1 M periodate reagent and thiobarbituric acid (TBA, 0.02 M), and the chromogenic product was extracted into butanol. Absorbance was measured at 548 nm using a spectrophotometer. Final quantification was performed against a standard calibration curve prepared from serial dilutions of KDO (ranging from 0 to 25 μM). The KDO concentrations in BEV preparations were expressed as μM/μg protein equivalent.

### 4.6. Compositional Analysis of *L. lactis* BEVs

#### 4.6.1. Carbohydrate

The total carbohydrate content of BEVs was quantified using the Anthrone assay, following the previously established protocol [17]. Briefly, an Anthrone reagent (HiMedia) was prepared in H_2_SO_4_ at a concentration of 2 g/L. A standard curve for carbohydrate content determination was established using various concentrations of D‐glucose (0–1 mg/mL). The carbohydrate content condensed with Anthrone was measured at 630 nm. BEV samples were processed similarly, and the carbohydrate content was quantified using dextrose as a standard (Supporting Figure S4C).

#### 4.6.2. Lipid

The lipid content was estimated by the standard sulfo‐phospho‐vanillin (SPV) assay. The artificial lipid vesicle (liposomes) was briefly synthesized using 1, 2‐ DOPC (Avanti Polar Lipids) at 1 mg/mL. A model liposome composed of DOPC in PBS was prepared using published methods [47]. In brief, 1.0 mL of methanol‐chloroform mixture (1:3 ratio) in a 2‐mL glass vial, and 1 mg of DOPC was solvated. The organic solvents were vaporized using a stream of nitrogen, followed by 12 h in a high vacuum to eliminate any remaining solvent. The dried DOPC was then refrigerated at 4°C for 3 h. The solution of PBS was then mixed with the lipid cake of dried DOPC, and the suspension was incubated for 1 h at 37°C with occasional vortexing. The resulting crude liposome suspension was sonicated in a bath‐type sonicator at 37°C for 30 min. Using this liposome solution, a standard for lipid content was prepared by the phospho‐vanillin assay with a slight modification of the previously published protocol [48]. Briefly, 200 μL of 96% sulfuric acid was added to either 40 μL of the standard liposome or 40 μL of the BEVs solution in PBS buffer. After brief vortexing, the open tubes were heated at 90°C using an electrical heating block (Cole‐Parmer) for 20 min. The tubes were cooled to 25°C by holding them at 4°C for at least 5 min. Subsequently, after dissolving vanillin (50 mg, Sigma, USA) in 17% phosphoric acid (50 mL), the phospho‐vanillin reagent was prepared. Then, 120 μL of phospho‐vanillin reagent was added to each tube and vortexed. After that, 200 μL of each solution was added to a 96‐well plate (Tarson, India), and the color was allowed to develop at 37°C for 1 h. After adding 80 μL of MilliQ water to each well, the absorbance was recorded using an Epoch 2 BioTek (USA) UV‐Spectrophotometer at 540 nm. A standard curve was generated (absorbance vs. concentration; fitted in a linear equation with *R* ^2^ = 0.99) and used as a reference to measure the total lipid concentration (Supporting Figure S4A).

#### 4.6.3. Protein Profile

Protein concentrations of isolated BEVs were quantified using the bicinchoninic acid (BCA) assay with a BCA protein assay kit (Thermo Fisher Scientific, USA) according to the manufacturer’s protocol, using bovine serum albumin (BSA) as a standard. For the protein profiling of BEVs, SDS–PAGE was performed using proteins from different subcellular fractions of *L. lactis*, following the previously mentioned method [49]. Briefly, *L. lactis* whole‐cell lysate (WCL) was prepared by incubating TES‐washed bacterial pellet (TES buffer: 10 mM Tris‐HCl [pH 8.0], 1 mM EDTA, 25% sucrose) in TES‐LMR buffer (lysozyme (1 mg/mL), mutanolysin (0.1 mg/mL), and RNase (0.1 mg/mL) for 1 h, at 37°C. The supernatant (cell wall fraction) and pellet (protoplast fraction) were collected after centrifugation at 2500 × *g* for 10 min. All the samples were dissolved in 4X sample buffer (4:1 v/v ratio), subjected to boiling for 5 min at 95°C, and loaded onto a 12% polyacrylamide gel.

#### 4.6.4. Proteomic Analysis of BEVs by LC‐ESI MS/MS

For mass spectrometric analysis, BEV samples were prepared using an in‐solution digestion procedure based on a previously published protocol [50]. Briefly, BEVs were subjected to overnight tryptic digestion at 37°C, using a protease‐to‐protein ratio of 1:20. Following the micro‐LC‐ESI MS/MS protocol, the resulting dried peptides were reconstituted in 10 μL of MS‐grade 0.1% formic acid in water. Peptides were then eluted through a microscale reverse‐phase C18 BEH column (Invitrogen) using a gradient of increasing concentrations of Solvent B [100% (v/v) acetonitrile with 0.1% formic acid] and decreasing concentrations of Solvent A [100% (v/v) MS‐grade water with 0.1% formic acid], supplemented with an isocratic flow of 100% MS‐grade isopropyl alcohol. Eluted peptides were analyzed using a Q‐TOF ion‐trap mass spectrometer, and mass spectra were acquired accordingly. Proteomic data were processed for bioinformatics analysis using Protein Lynx Global SERVER™ (PLGS) Version 3 (Waters), referencing the *L. lactis* proteome from the UniProt database, including reviewed and unreviewed entries. The subcellular localization of identified proteins was predicted using PSORTb Version 3.0.3 (https://www.psort.org/psortb/).

#### 4.6.5. Muramic Acid (for PGN)

As an indirect method for estimating the PGN content of BEVs, we performed a standard muramic acid assay. This involved hydrolyzing the muramic acid to lactic acid, then degrading it to acetaldehyde, and subsequently forming a complex with p‐hydroxy diphenyl (PHD). The amount of hydroxydiphenyl in the sample was then measured colorimetrically (*A*_560_ nm) using previously published methods [51]. Briefly, using 1 M NaOH, a volume containing muramic acid (∼5–20 μg) was created, up to 0.5 mL, in 10‐mL round‐bottom flasks from a 1 mg/mL stock solution of muramic acid. The mixtures were incubated at ∼ 38°C for 30 min, followed by the addition of 500 μL of 0.5 M H_2_SO_4_ and 5 mL of concentrated H_2_SO_4_ added dropwise to prevent overheating. The flask was placed inside an electric kettle for ∼5 min under boiling water. After 5 min, the flasks were cooled at room temperature, and 50 μL aqueous solution of CuSO_4_ was added, followed by 100 μL PHD ethanolic solution under continuous stirring for 30 min in two spells. Finally, the absorbance was recorded at 560 nm using an Epoch 2 BioTek UV‐Spectrophotometer. A standard curve was generated (absorbance vs. concentration; fitted in a linear equation with *R* ^2^ = 0.99) and used as a reference to measure the concentration (Supporting Figure S4B).

### 4.7. *In Vitro* Functionality of *L. lactis* BEVs

#### 4.7.1. The Fusogenic Potential of BEVs by FRET Assay

Artificial vesicles (liposomes) were prepared following the previously published methods with slight modifications to assess the fusogenic potential of BEVs [16]. Briefly, fluorophore‐probed liposomes were prepared using DOPC/DOPE/DOPS/Chol/NBD‐PE/Rh‐PE at a molar ratio of 44/27/6/20/1.5/1.5, while unprobed vesicles were prepared using DOPC/DOPE/DOPS/Chol at a molar ratio of 44/30/6/20. The lipids at these ratios were dissolved in a chloroform–methanol (3:1) mixture, and the solvent was evaporated using a nitrogen stream to create a thin lipid film, which was further vacuum‐dried overnight. The next day, the lipid film was hydrated at 37°C for 3 h and vortexed every 15 min, using 20 mM HEPES buffer containing 150 mM NaCl at pH ∼7.4. Small unilamellar vesicles (SUVs) were formed using probe sonication (Hielscher Ultrasound Technology, Germany) and were extruded 19 times through 50‐nm polycarbonate filters. The size of the resulting vesicles was measured at ∼60 nm.

To examine the fusion kinetics of BEVs with probed liposomes, we performed a FRET assay following the previously established method with slight modifications [47]. The fusion kinetics were tracked using FRET between NBD (donor) and Rhodamine B (Rh‐acceptor), which are covalently attached to the DOPE headgroup. For this, fluorophore‐doped liposomes (DOPC/DOPE/DOPS/Chol/NBD‐PE/Rh‐PE) were mixed with unlabeled BEVs at a 1:20 molar ratio. A fusion experiment was carried out in a 20 mM HEPES buffer containing 150 mM NaCl and 2 mM CaCl_2_ at pH 7.4. The emission intensity (at 535 nm) of the donor (NBD‐PE) was monitored with an excitation wavelength of 460 nm (Hitachi F‐7000 spectrofluorometer, 5‐nm slit).

The percentage of membrane fusion was calculated using the following equation:

(1) Percentage of membrane fusion=Ft−F0F∞−F0×100,

where *F*_0_, *F*_*t*_, and *F*_*∞*_ are the fluorescence intensities at the initial time, time t, and time ∞ (measured in the presence of Triton X‐100, which induces nearly 100% FRET dilution by disrupting the membrane), respectively.

#### 4.7.2. *In Vitro* Cellular Uptake of BEVs

To facilitate intracellular tracking, *L. lactis* BEVs were first labeled with 1,1′‐dioctadecyl‐3,3,3′,3′‐tetramethylindocarbocyanine perchlorate (Dil‐C_18_) (Sigma‐Aldrich) as per a previously published method with minor modifications [52]. Briefly, BEVs were dissolved in PBS containing NaCl concentration to ∼20 mM, mixed with ∼2 μM Dil‐C_18_ (lipophilic dye), followed by sonication in a bath‐type sonicator (50/60 Hz) for 15 min, followed by 30‐min incubation at 37°C. A small volume of 10x PBS was added to increase the NaCl concentration to ∼150 mM. The mixture was finally filtered through a 0.22‐μm syringe filter (Whatman, UK) to remove dye aggregates, and only Dil‐C_18_–labeled BEVs were collected.

To investigate the cellular uptake of Dil‐C_18_–labeled BEVs, we used murine macrophage cells (J774A.1) and human HeLa cells. The fluorescent‐labeled BEVs were incubated with the indicated cell line for various time points (15 min, 3, 6, 12, and 24 h). After incubation, cells were washed with 1X PBS, followed by fixation with 4% paraformaldehyde (PFA) for 20 min. Following two PBS washes, the nucleus was stained with 4′,6‐diamidino‐2‐phenylindole (DAPI) (Sigma). Finally, the cells were mounted on a glass slide using a Vectashield mounting medium (Vector Laboratories, Inc., USA) and subjected to confocal laser microscopy (CLSM) using a Leica SP8 microscope (Leica Microsystem, Wetzlar, Germany) with an HC‐PL‐APO‐CS2 63×/1.4 oil immersion lens. The fluorescence signal was recorded at 405‐nm laser (for DAPI‐stained nuclei) and 552‐nm laser (for Dil‐C_18_ tagged BEVs). The image analysis was performed using Fiji software.

#### 4.7.3. Mechanism of Cellular Uptake

To study the mechanism of cellular uptake of BEVs by HeLa (as nonimmune cells) and J774A.1 (as immune cells), cells were pretreated with a specific chemical inhibitor targeting the endocytic pathway, specifically dynamin, following a previously described method [53]. In brief, cells were pretreated with 80‐μM dynasore (inhibitor clathrin‐caveolin–mediated endocytosis [CME]) for 1 h at 37°C under 5% CO_2_, followed by treating the cells with Dil‐C_18_ labeled BEVs (20 μg/mL = ∼2 × 10^10^ particles/mL) for 6 h. After incubation, cells were washed with PBS and the fluorescence of extracellular Dil‐C_18_‐BEVs was quenched using 0.025% trypan blue before fixation in 4% PFA. Following fixation, actin filaments of the cell membrane were stained with phalloidin 647 (Abcam, USA), while the nuclei were stained with DAPI. Imaging of the cells was performed as described in the previous section. The MFI of untreated cells incubated with Dil‐C_18_‐BEVs served as the control, and its MFI was considered 100%. The percentage of Dil‐C_18_‐BEVs uptake by the target cells pretreated with the inhibitor was calculated following the previously mentioned formula [16].

### 4.8. Assessing the Antiproliferative Effect of Drug‐Loaded BEVs Against Cancer Cells

#### 4.8.1. Encapsulation of Doxorubicin Into BEVs

To assess the potential of BEVs as an anticancer drug carrier, we used doxorubicin, a known anthracycline that restricts cancer cell growth by blocking Topoisomerase 2. Doxorubicin (Sigma‐Aldrich) was loaded into freshly prepared BEVs by the electroporation method [54]. Briefly, 500 μg/mL of BEVs dissolved in 100 μL PBS was mixed with 250 μg/mL of doxorubicin (in 200 μL, prepared in sterile MilliQ) and cold electroporation buffer (100 μL, 10% glycerol, 500 mM sucrose, pH∼7). The mixture was subjected to electroporation in a Gene Pulser II Electroporator (Bio‐Rad, USA). Postelectroporation, BEVs were incubated at 37°C for 30 min to allow membrane recovery, followed by several PBS washes using ultracentrifugation (200, 000 × *g*) to remove free doxorubicin. Finally, the sample was filtered through a 0.22‐μM syringe filter. The concentration of encapsulated doxorubicin was quantified by recording the fluorescence intensity using a BioTek Cytation 5 multimode reader (excitation at 480 nm and emission at 590 nm). An optimal electroporation condition was found to be the following: voltage: 250 V, resistance: 350 μF, and pulse: single pulse. The percentage (%) encapsulation was calculated as per the following equation:

(2) % of Encapsulation= Total concenctration of DOX in BEVDOX−BEVDOX after electroporationInitial concentration of DOX used during encapsulation×100.

#### 4.8.2. *In Vitro* Release Kinetics of Dox From BEV_DOX_

To assess the drug release profile of doxorubicin from drug‐loaded BEVs, we performed dialysis‐based release kinetics in PBS at pH 7.4 (as physiological buffer) and pH 5.5 (to mimic the acidic tumor microenvironment). In brief, 2.0 mL of BEV_DOX_ dissolved in PBS was poured into a dialysis bag (50‐kDa cutoff) and incubated in 100 mL of PBS buffer (pH 7.4 and 5.5) in the presence of 10 μM H_2_O_2_ at 37°C with constant stirring. At every 1‐h interval, 200 μL of the buffer was collected, and emission was monitored at 595 nm by fluorescence spectroscopy to measure the Dox released into the external buffer. The dialysate was then poured back into the beaker. A percentage of cumulative drug release was calculated and plotted using GraphPad Prism 8 as per the following equation:

(3) Percentage of cumulative drug release=Emmission of DOX in bufferEmission of Dox after full release into the buffer ×100.

#### 4.8.3. *In Vitro* Clonogenic Assay

*In vitro* cell survival probability was assessed by clonogenic assay to determine the effect of Dox‐loaded BEVs (BEV_DOX_) on the ability of a single cancer cell to form a colony [55]. Briefly, MCF‐7 cells (∼1 × 10^4^ cells/well in six‐well plates) were grown overnight. The following day, cells were either treated with free DOX (10 ng/mL) or treated with DOX‐loaded BEV (equivalent concentration), or left untreated for 48 h. Next, a fresh medium was added, and cells were allowed to grow for 8 days with intermediate changes in medium (every 3 days). After forming the colonies (at least 50 cells/colony), cells were fixed with 2.5% glutaraldehyde and staianed with 5% crystal violet, and images were acquired. The stained colonies were dissolved in DMSO, and absorbance was measured at 595 nm using a Bio‐Tek multiplate reader.

### 4.9. Cell Cycle Analysis

Cell cycle analysis was performed using PI/RNase staining by flow cytometry according to a previously established protocol [56]. Briefly, human breast cancer cells (MCF‐7) were seeded at 1 × 10^4^ cells/well in 24‐well plates at 5% CO_2_ and 37°C until they reached 80% confluence. The cells were treated for 48 h with either free Dox (0.6 μg/mL) or Dox‐loaded BEV (BEV_DOX_; 0.6 μg/mL). Next, following PBS wash, cells were trypsinized with a 0.25% trypsin‐EDTA solution (Gibco) and resuspended in prechilled 70% ethanol to fix and permeabilize the cells overnight at 4°C. Subsequently, after decanting the fixative, cells were washed twice with PBS and stained with PI (1 μg/mL) and RNAase‐A (50 μg/mL) solution for 10 min at 37°C. After two additional washes with PBS, the sample was resuspended in 500 μL PBS and subjected to flow cytometric analysis (BD‐LSR Fortessa).

### 4.10. Analysis of Apoptosis by Annexin‐V/PI Staining and Flow Cytometry

The Annexin‐V flow cytometric assay was used to detect PS on early apoptotic cells for a comparative assessment of the apoptotic effect of free Dox or Dox‐loaded BEV treatment on MCF‐7 cells. For this, we used the Annexin‐V FITC apoptosis detection kit (BD Biosciences) as per the manufacturer’s protocol. Briefly, MCF‐7 cells were seeded on a 6‐well plate and incubated up to 80% confluency. Next, cells were treated with free Dox (0.6 μg/mL) or BEV_DOX_ (equivalent drug concentration) for 48 h in the DMEM supplemented medium at 37°C under 5% CO_2_. Following incubation, cells were washed with PBS and subjected to trypsinization. After one PBS wash, the cells were resuspended in the binding buffer. Annexin‐V FITC conjugate and PI solution were added to cells in dark for 15 min at RT. Finally, the percentage of apoptotic cells was analyzed immediately using BD‐LSR Fortessa flow cytometer with laser excitation 490 nm for Annexin‐V and 550 nm for PI.

### 4.11. Effect of BEV_DOX_ on Cell Migration

To determine the antiproliferative effect of BEV_DOX_, a standard cell migration assay was performed by “scratch assay” [57]. Briefly, the MCF‐7 cells were seeded in a six‐well plate (3 × 10^5^ cells/well) with complete DMEM and incubated at 37°C, 5% CO_2_ to form a monolayer. A sterile P20 pipette tip was used to create a single scratch on the monolayer, followed by washing with PBS to remove debris or loose cells. The cells were treated with either Dox (0.6 μg/mL) or BEV_DOX_ (at equal concentrations), and cells without treatment served as controls. The cell migration to cover the scratch was monitored for 48 h, and bright‐field microscopic images were captured at regular intervals.

### 4.12. AO/PI Double Staining

The apoptotic effect of doxorubicin was further analyzed by AO/PI double staining, where AO binds to the DNA of all cells and emits green fluorescence and PI marks only dead cells. In brief, MCF‐7 cells were seeded at 1 × 10^5^ cells/mL in 12‐well plates and treated with DOX (0.6 μg/mL), BEV_DOX_ (equivalent), or left untreated for 36 h. After incubation, cells were trypsinized and washed with PBS. Next, cells were stained with 1 μg/mL AO (HiMedia) and 1 μg/mL PI (Sigma) for 2 min and washed once with PBS. Approximately 5 μL of culture was cast on a glass slide and assessed under IX‐83 epifluorescence microscope using a 63X oil immersion lens. The acquired images were further analyzed using Fiji software.

### 4.13. Antimicrobial Effect of Drug‐Loaded BEV Against MRSA, *C. jejuni*, and *C. albicans*

#### 4.13.1. Encapsulation of Gentamicin Into BEVs (BEV_GEN_)

We used gentamicin as a standard antibiotic to see the effect of antibiotic‐loaded BEVs against MRSA and *C. jejuni*. The gentamicin (400 μg/mL, HiMedia, India) was encapsulated into BEVs (BEV_GEN_; 1 mg/mL) using electroporation with a single pulse at 250 V and 350 μF, followed by incubation at 37°C for 30 min (Figure 5(a): A). Next, after several washes through ultracentrifugation with PBS buffer and filtration (0.22‐μm syringe filter), the sample was stored at −20°C for future use. The efficiency of gentamicin encapsulation was quantified using a ninhydrin colorimetric assay, as mentioned earlier, by measuring the ninhydrin–gentamicin complex with minor modifications [58, 59]. Briefly, a known amount of gentamicin was dissolved in 1X PBS buffer (pH 7.4), and a series of dilutions (2‐fold) were made. For ninhydrin, approximately 1.25% (w/v) solution was prepared by dissolving ninhydrin in the PBS (pH 7.4) and ∼75 μL of the ninhydrin solution was mixed with the different dilutions of gentamicin, maintaining a total volume of 500 μL. Next, the mixture of gentamicin and ninhydrin was heated to 95°C for 15 min and cooled in an ice bath. Then, the solutions were placed into a 96‐well plate, and absorbances were recorded at 418 nm using a BioTek microplate reader. To determine the amount of gentamicin encapsulated in BEVs, a standard curve was generated (absorbance vs. concentration; fitted in a linear equation with *R* ^2^ = 0.9986) and used as a reference. Finally, the encapsulation efficiency (%) was determined using the following equation:

(4) % of Encapsulation= Total concentration of gentamicin in BEV after electroporationInitial concentration of gentamicin used during encapsulation×100.

### 4.14. Antifungal Effect of Amphotericin B (AmB) Loaded BEV Against *C. albicans*

#### 4.14.1. Encapsulation of AmB Into BEVs (BEV_AmB_)

As a common antifungal, Amphotericin B (MycoFlu, Jolly Healthcare, India, clinical grade) was encapsulated into BEVs using the passive loading method following a previously published protocol with minor modifications (Figure 6(a)) [60]. Briefly, BEVs (1 mg/mL of total protein content) were incubated with AmB (250 μg/mL) in PBS and then sonicated in a bath‐type sonicator (50/60 Hz) for 15 min, followed by recovery at 37°C for 30 min. The free (unloaded) drugs were separated by using size exclusion column (SEC) chromatography with Sephadex G50 bead as the stationary phase and 1X PBS buffer (pH 7.4) as the mobile phase. After passing through the column, a few drops of the eluents were collected, and absorbance was checked at 328 nm (*A*_328_). The eluents with higher absorbance were collected and passed through a 0.22‐μM membrane filter. To quantify AmB encapsulated in the BEVs, a standard curve of AmB was generated (absorbance vs. concentration; fitted in a linear equation with *R* ^2^ = 0.9980) and used as a reference. Finally, the encapsulation efficiency (%) was determined using the following equation:

(5) % of Encapsulation= Total concentration of AmB in BEV after electroporationInitial concentration of AmB used during encapsulation×100.

### 4.15. Antibacterial and/or Antifungal Efficacy of Drug‐Loaded BEVs by Solid and Liquid Phase Assay

To evaluate the antibacterial and antifungal effect of gentamicin or AmB‐loaded BEV, MRSA, *C. jejuni*, or *C. albicans* cultures were evenly spread on agar plates: LB agar (HiMedia) for MRSA, Campylobacter selective black agar medium (HiMedia), and M‐BCG yeast and mold medium agar (HiMedia) for *C. albicans*.

#### 4.15.1. Solid‐Phase Assay

Equal‐sized wells were punched in the plate and filled with varying concentrations of either free AmB or gentamicin (100 μL maximum volume/well) or drug‐loaded BEVs, followed by incubation at 37°C under static conditions for 24 h. Finally, the diameter of the inhibition (which appeared to be a halo) surrounding the wells was measured, and plate images were acquired using a Bio‐Rad Gel imaging and documentation unit. The diameters of the zone of inhibition were plotted to assess diffusion and concentration‐dependent inhibition, and the minimum inhibitory concentration (MIC) was determined using linear fitting. All experiments were performed in triplicate and presented as mean ± SE.

#### 4.15.2. Liquid‐Phase Assay

For the liquid‐phase assay, we employed the microbroth dilution method as described in a previously published method [61]. Briefly, the MIC was determined using the microbroth dilution method for both MRSA and *C. jejuni* (TGH9011) in LB broth and MH broth, respectively. Briefly, free gentamicin and gentamicin‐loaded BEV (BEV_GEN_) were subjected to 2‐fold dilution starting from 12 μg/mL to 12 ng/mL on a 96‐well plate and incubated with each of the bacteria (either MRSA or *C. jejuni*, OD‐0.2) under optimal conditions for 24 h. Finally, the absorbance (*A*_600_) was recorded for each well using a multimode plate reader (BioTek), and the percentage of bacterial growth (%) was calculated to determine the bacterial growth as well as the MIC_50_.

#### 4.15.3. SEM Image Processing

To assess the morphological changes and structural deformities of *C. jejuni*, *S. aureus*, and *C. albicans* cells treated with the respective antibiotics (either in free form or with BEVs), the cells were processed for FESEM analysis according to the methods discussed in the previous section. Untreated bacteria were kept as a control group (untreated) for 12 h.

### 4.16. Statistical Analysis

The GraphPad Prism statistical software (Version 8) was used for graphical presentations and data analysis. The diameters of SEM and TEM images were examined using ImageJ software. The D’Agostino–Pearson test and Shapiro–Wilk test were performed to confirm the normal distribution. The Student’s *t* test (two‐tailed, unpaired) was used to compare the significance between two experimental groups, and one‐way ANOVA was used to compare the significance among multiple experimental groups. The values of ^∗^ *p* ≤ 0.05 and ^∗∗^ *p* ≤ 0.01 were considered statistically significant.

**Nomenclature**
BEV: Bacterial extracellular vesicle
LAB: Lactic acid bacteria
GRAS: Generally recognized as safe
FRET: Förster resonance energy transfer
MFI: Mean fluorescence intensity
DOX: Doxorubicin
MRSA: Methicillin‐resistant *Staphylococcus aureus*
CLSM: Confocal laser scanning microscopy
EVs: Extracellular vesicle
OMVs: Outer membrane vesicle

## Ethics Statement

The authors have nothing to report.

## Consent

The authors read the manuscript and gave their consent for the publication.

## Conflicts of Interest

The authors declare no conflicts of interest.

## Author Contributions

Sushmita Das performed the experiments, analyzed the data, and wrote the manuscript. Subhadeep Gupta performed the experiments, analyzed the data, and wrote the manuscript. Subrata Das performed the membrane fusion study under the supervision of PKT. Afruja Khan performed *in vitro* cellular uptake pathway assay. Pradip Kumar Tarafdar analyzed the membrane fusion data and wrote the respective section. Amirul Islam Mallick conceived the study, analyzed the data, and wrote the manuscript.

## Funding

Amirul Islam Mallick acknowledges the IISER Kolkata Institutional Fund (Academic Research Fund) and the Core Research Grant (CRG)—Anusandhan National Research Foundation (ANRF), Government of India (Grant No. CRG/2022/003383), for supporting this research. Pradip Kumar Tarafdar thanks the Science and Engineering Research Board (SERB) Core Research Grant (Grant No. CRG/2022/008554) for the financial support.

## Supporting Information

Supporting Information Description: This supporting information provides additional information and data supporting the study presented in the main manuscript. It includes additional data and images related to the morphological, compositional, and biophysical characterization of BEVs under different treatment conditions. A list of proteins identified through mass spectrometric analysis of BEVs. This supporting information is essential for understanding the biophysical, compositional, and functional (antimicrobial and anticancer) properties of the BEVs used in this study to support the conclusions drawn in the main text.

Supporting Information Figures and Tables: Figures and tables related to the supporting information are provided as separate files within the review system. These files are labeled as Figure S1 (the zeta‐potential [*ζ*] of BEVs naturally secreted by *L. lactis*); Figure S2 (effect of ampicillin and chloramphenicol on *L. lactis* growth profile); Figure S3 (comparative analysis of BEVs uptake by human HeLa and murine J774A.1 cells pretreated with dynasore); Figure S4 (standard curve generated using relevant standards for SPV assay [for lipid quantification], (B) muramic acid assay [for PGN quantification] (C), and Anthrone assay [for carbohydrate quantification]); Figure S5 (determining the IC_50_ of empty BEVs and doxorubicin‐HCL [μg/mL] by cell viability assay for human MCF‐7 cells); Figure S6 (encapsulation of doxorubicin–HCL [DOX], into BEVs [BEV_DOX_] isolated from ampicillin [0.1 μg/mL]‐treated *L. lactis* culture); Figure S7 (generation of standard curve correlating diameter of zone of inhibition [mm] with the known concentration of gentamicin against *S. aureus* and *C. jejuni*; and Amphotericin B against *C. albicans*). Table S1 (list of proteins detected by mass spectrometry analysis of *L. lactis* BEVs); Table S2 (analysis of batch‐to‐batch variability in BEV yield with respect to the total protein content).

Note: The supporting information figures and tables are not included in the main manuscript file to maintain the clarity and conciseness of the presentation.

## Supporting information


**Supporting Information** Additional supporting information can be found online in the Supporting Information section.

## Data Availability

The data that support the findings of this study are available within the article and its supporting information.
