# Supplementary material for: Bacterial Extracellular Vesicles (BEVs) Derived From Lactococcus lactis as Multimodal Drug Delivery Platforms
Source: Int J Biomater. 2025 Dec 31;2025:3141223. doi: 10.1155/ijbm/3141223 (PMC12755118; doi:10.1155/ijbm/3141223)
Supplement: Supplementary file 1 — Supporting Information Additional supporting information can be found online in the Supporting Information section. [file IJBM-2025-3141223-s001.docx]

**Supporting Information**

**Bacterial Extracellular Vesicles (BEVs) Derived From *Lactococcus lactis* as Multimodal Drug Delivery Platforms**

Sushmita Das^1^, Subrata Das^2^, Subhadeep Gupta^1^, Afruja Khan^1^, Pradip Kumar Tarafdar^2^* and

Amirul Islam Mallick^1^*

**Author’s affiliations:**

^1^ Department of Biological Sciences, Indian Institute of Science Education and Research Kolkata, Mohanpur, Nadia, West Bengal, PIN: 741246, India.

^2^ Department of Chemical Science, Indian Institute of Science Education and Research Kolkata, Mohanpur, Nadia, West Bengal, PIN: 741246, India.

***Corresponding Authors’ Information:**

**^1^** Dr. Amirul Islam Mallick, Professor, Department of Biological Sciences, Indian Institute of Science Education and Research Kolkata, Mohanpur, Nadia, West Bengal, PIN: 741246, India. Ph. 91-33-61360022-Ext 1221, E-mail: [amallick@iiserkol.ac.in](mailto:amallick@iiserkol.ac.in); ORCID: 0000-0002-2265-9856

**^2^** Dr. Pradip Kumar Tarafdar, Associate Professor, Department of Chemical Sciences, Indian Institute of Science Education and Research Kolkata, Mohanpur, Nadia, West Bengal, PIN: 741246, India. E-mail: tarafdar[@iiserkol.ac.in](mailto:amallick@iiserkol.ac.in); ORCID: 0000-0002-1059-950X

**Figure. S1: The zeta potential (ζ) of BEVs naturally secreted by *L. lactis*.**

**Figure S1:** The zeta potential (ζ) of naturally secreted BEVs from *L.lactis* (NZ9000) was evaluated using Dynamic Light Scattering (DLS) on a Malvern Zetasizer Nano ZS instrument (USA). The sample was prepared in MilliQ at a 1:50 dilution, followed by ultrasonic sonication for 15 min in a bath-type sonicator to ensure dispersion. The measurements were performed at 25°C using a capillary cell, and data were acquired with 10 technical replicates. The Zeta potential of BEVs showed a net negative surface charge of approximately -23mV, indicating an overall anionic surface property of BEVs.

**Figure S2: Effect of Ampicillin and Chloramphenicol on *L. lactis* growth profile.**

**Figure S2: Effect of Ampicillin and Chloramphenicol on *L. lactis* growth profile.** A micro broth dilution method was performed using serial 2-fold dilutions of varying antibiotic concentrations to assess the Minimum Inhibitory Concentration-50 (MIC_50_). Following a 24 h incubation, bacterial growth was measured by recording the absorbance at 600 nm (A_600_). The percent growth was calculated relative to the control bacterial culture (no treatment) and plotted against drug concentration. MIC_50_ values Chloramphenicol (A) and Ampicillin (B) were calculated as ~3.9 µg/mL and ~0.35 µg/mL, respectively, by fitting with a nonlinear regression equation using GraphPad Prism-8 software.

**Figure S3: Scanning electron micrograph (SEM) of BEVs isolated from Ampicillin-treated *L.lactis* (BEV_Amp_)**


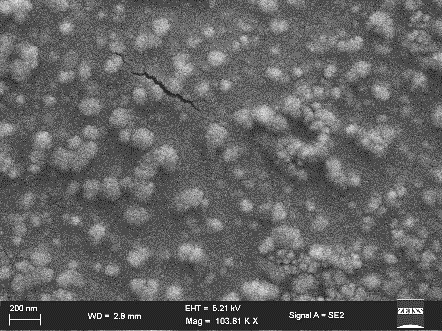

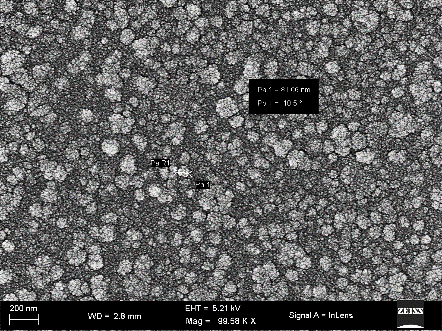

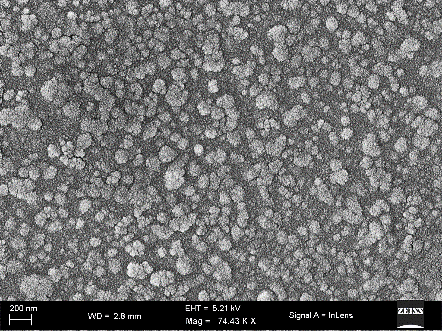


**Figure S3: The structural and morphological characterization of BEV_Amp_** (Ampicillin-treated *L.lactis* culture, 0.1µg/mL) was assessed by Field Emission Scanning Electron Microscopy (FESEM), showing vesicular and heterogeneous distribution.

**Figure S4: Standard curve generated using standards for (A) Sulpho-phospho vanillin assay (SPV assay) (for Lipid quantification), (B) Muramic acid assay (for peptidoglycan quantification), and (C) Anthrone assay (for carbohydrate quantification).**

**Figure S4:** Liposome made up of DOPC was used to prepare a standard curve for SPV-lipid quantification based on a colorimetric assay (A).To evaluate peptidoglycan content in BEVs, muramic acid, one of the main constituents of peptidoglycan, was quantified using a muramic acid assay. A standard curve was generated with 5-20 µg of commercially available muramic acid (B). To quantify the carbohydrate content, a standard curve of D-glucose/dextrose (0-1 mg/mL) was prepared using the Anthrone colorimetric assay (C).

**Figure S5: Determining the cytotoxicity (IC_50_) of empty BEVs and Doxorubicin-HCL (µg/mL) by standard cell viability assay (MTT assay) in human MCF-7 cells**

**Figure S5:** To evaluate the cell cytotoxicity of only BEVs and Doxorubicin-HCL (DOX), a standard MTT assay was performed at 48 h post-treatment with BEVs (80µg/mL to 150ng/mL protein concentration) and different Doxorubicin concentrations (50µg/mL to 0.76 ng/mL) using MCF-7 cells seeded on a 96-well plate to 70% confluence. Following incubation, cells were washed with PBS and incubated with 3-(4, 5-dimethylthiazol-2-yl)-2, 5-diphenyltetrazolium Bromide (MTT, HiMedia, India) at 1.0 mg/mL in DMEM media and incubated for 3 h at 37°C. Formazan crystals formed were dissolved in DMSO, and the absorbance was measured at 595nm (A_595_). The percent cell viability was calculated and fitted to a nonlinear equation, showing that empty BEVs were not cytotoxic (A), while Doxorubicin showed an IC_50_ value of ~2.0 µg/mL (B).

**Figure S6: Encapsulation of Doxorubicin-HCL (DOX) into BEVs (BEV_DOX_) isolated from Ampicillin (0.1µg/ml) treated *L. lactis* culture.**

**Figure S6.** **Optimization of electroporation conditions for drug encapsulation in BEVs.**
To assess the optimal conditions for encapsulating BEVs with Dox by electroporation, BEVs were subjected to different electroporation conditions: voltage, 250 V, 470 V, and 800 V, with a resistance of 350 µF, followed by recovery at 37°C for 30 min. Following electroporation and several PBS washes, the fluorescence signal of drug-encapsulated BEVs (250 V, 470 V, 800 V, and buffer control) was measured **(A)**, suggesting that electroporation at 250 V resulted in maximum Dox encapsulation into BEVs. To further confirm the Dox loading, spectral analysis of the BEVs (250V) sample after Triton X-100 (10%) treatment confirmed the complete release of the encapsulated drug **(B)**. Biophysical characterization of BEV_DOX_ (post-electroporation) was evaluated by assessing size distribution using nanoparticle tracking analysis (NTA) **(D)**. Furthermore, light scattering (532 nm) and fluorescence spectrum (excitation: 532 nm, emission: 565 nm) analysis by NTA showed that ~54% of BEV_DOX_ were positively labeled with DOX **(C)**. Transmission electron microscopy (TEM) and field emission scanning electron microscopy (FE-SEM) images of BEV_DOX_, confirming that electroporation did not alter vesicle morphology **(E, F).** The plot represents the encapsulation efficiency of Doxorubicin into BEVs **(G)**.

**Figure S7: Generation of standard curve correlating the diameter of zone of inhibition (mm) created by known concentration of Gentamicin against *S. aureus* (MRSA) and *C. jejuni*; and Amphotericin-B against *C. albicans*.**

**A.**

**B.**

**C.**

**Figure S7. Standard curve based on the diameter of the zone of inhibition (mm)** on solid phase Agar-well diffusion method for MRSA (A), *C. jejuni* (B)*, and C. albicans* (C)*.* The red and purple lines on the standard indicate the corresponding diameter of zone inhibition caused by Gentamicin-loaded BEVs against MRSA **(A).** The orange and sky blue lines on the standard indicate the corresponding diameter of zone inhibition caused by Gentamicin-loaded BEVs against *C. jejuni* **(B).** The green line on the standard curve indicates the corresponding diameter of zone inhibition caused by Amphotericin-B loaded BEVs against *C. albicans* **(C).**

**Table S1. List of Proteins detected by mass spectrometry analysis of *L. lactis* BEVs.**

| **Accession** | **Description** | **mW (Da)** | **pI (pH)** | **PLGS Score** | **Peptides** | **Theoretical Peptides** | **Coverage (%)** |
| --- | --- | --- | --- | --- | --- | --- | --- |
| A0A0M2ZTQ2 | CHAP domain-containing protein | 33984 | 9.394 | 2083.303 | 4 | 18 | 34.6535 |
| A0A1V0PFV5 | Surface antigen | 31653 | 9.252 | 2083.303 | 4 | 19 | 37.234 |
| A0A2Z5Z5S3 | Immunogenic secreted protein-like protein | 33931 | 9.3237 | 2083.303 | 4 | 18 | 34.6535 |
| A2RJP5 | Immunogenic secreted protein homolog | 34014 | 9.394 | 2083.303 | 4 | 18 | 34.6535 |
| A0A0M2ZUN6 | NHLM bacteriocin ABC transporter_ peptidase/ATP-binding protein | 38593 | 6.6167 | 625.4531 | 6 | 17 | 38.0814 |
| A0A895ICU1 | Glycosylhydrolase- family 25 | 38643 | 6.6167 | 625.4531 | 6 | 17 | 38.0814 |
| A2RIJ0 | Peptidase_C39_2 domain-containing protein | 38593 | 6.6167 | 625.4531 | 6 | 17 | 38.0814 |
| A0A1V0NSX6 | Cell surface protein | 38577 | 6.6167 | 609.7747 | 5 | 17 | 28.1977 |
| A0A896T867 | Peptidase_C39_2 domain-containing protein | 44688 | 8.2573 | 573.2092 | 10 | 26 | 40.4938 |
| A0A0M2ZUS3 | Cell wall surface anchor protein | 44711 | 8.2603 | 573.2092 | 10 | 26 | 40.4938 |
| A0A1V0PF30 | Glycosylhydrolase family 25 | 43417 | 6.7222 | 570.188 | 9 | 27 | 37.0558 |
| A0A2Z5Z202 | Glycosylhydrolase_ family 25 | 43430 | 6.7222 | 570.188 | 9 | 27 | 37.0558 |
| A2RIJ1 | Peptidase_C39_2 domain-containing protein | 43425 | 6.791 | 570.188 | 9 | 27 | 37.0558 |
| A0A1V0NSY9 | Glycosyhydrolase | 43402 | 6.7222 | 570.188 | 9 | 27 | 37.0558 |
| A0A895IBV5 | Glycosylhydrolase- family 25 | 43436 | 6.7222 | 535.907 | 8 | 27 | 30.7107 |
| A0A6I2IAP8 | Glyceraldehyde-3-phosphate dehydrogenase | 35797 | 5.4712 | 1979.859 | 10 | 22 | 36.9048 |
| A0A6N3KEH7 | Glyceraldehyde-3-phosphate dehydrogenase | 35811 | 5.4712 | 1974.493 | 8 | 22 | 28.869 |
| A2RP55 | Glyceraldehyde-3-phosphate dehydrogenase | 35797 | 5.4712 | 1974.493 | 10 | 22 | 36.9048 |
| A0A0M2ZRZ8 | Glyceraldehyde-3-phosphate dehydrogenase | 35797 | 5.4712 | 1974.493 | 10 | 22 | 36.9048 |
| A0A7X2KWV9 | VWA domain-containing protein | 161008 | 4.3872 | 643.3686 | 15 | 114 | 21.1445 |
| A0A6I2HGG0 | Metal-independent alpha-mannosidase | 50202 | 4.7769 | 687.1613 | 8 | 39 | 42.4594 |
| A0A161UKC3 | Uncharacterized protein | 50180 | 4.812 | 649.4551 | 6 | 39 | 34.8028 |
| A0A895IHA8 | HTH luxR-type domain-containing protein | 33221 | 4.7637 | 592.4523 | 16 | 200 | 40.5109 |
| A0A896TAI7 | Cell surface protein | 37926 | 9.7119 | 158.404 | 5 | 24 | 24.0356 |
| A0A1V0P9K2 | Cell surface protein | 37812 | 9.7119 | 158.404 | 5 | 24 | 24.1071 |
| A0A7X2GUR9 | MATE family efflux transporter | 47127 | 10.314 | 150.15 | 7 | 21 | 50.5827 |
| A0A1V0P9K2 | Cell surface protein | 37812 | 9.7119 | 158.404 | 5 | 24 | 24.1071 |
| A0A896TBG7 | Surface antigen | 11067 | 9.2534 | 650.1368 | 3 | 5 | 55.5556 |

**Table S2:** **Analysis of batch-to-batch variability in BEVs yield with respect to total protein content**

| **Experiments** | **Standard deviation (SD)**  **(Total protein content of BEVs)**  **(w/o Antibiotic)**  **(A)** | **Coefficient of variation (n=6)** | **Experiment** | **Standard deviation (SD)**  **(Total protein content of BEVs)**  **(treated with Ampicillin)**  **(B)** | **Coefficients of variation (n=6)** |
| --- | --- | --- | --- | --- | --- |
| Biological replicate-1 (n=3) | 0.358490005 | **13.61** | Biological replicate-1 (n=3) | 0.885738 | **5.49** |
| Biological replicate-2 (n=3) | 0.730638 |  | Biological replicate-2 (n=3) | 0.088285 |  |

**Batch-to-batch variability:** To determine batch-to-batch variability in BEV yield, we compared the variance in the total protein content of isolated BEVs from two biological replicates (each of which comprises three technical replicates, n = 6). For this, BEVs isolated from bacterial culture (in 150 mL) in the absence **(A)** or presence of 0.1 µg/mL ampicillin (0.1 µg/mL) **(B)** were taken into account. The table showing the Standard deviation (**SD**) and Coefficients of Variation (**CV)** using the following equation for each data set (**A** and **B**): **CV = (Standard Deviation / Mean) × 100.** The calculated CV was found to be within the limit, hence the observed variability is acceptable.

**Table S3. Comparative analysis of BEVs uptake (%) by human HeLa and murine J774A.1 cells pre-treated with dynasore.**

| **Chemical inhibitor** | **Human HeLa cells** | | **Murine J774A.1** | |
| --- | --- | --- | --- | --- |
|  | **% uptake of BEVs** | **% reduction of BEVs uptake** | **% uptake of BEVs** | **% reduction of BEVs uptake** |
| Dynasore | 46.81±0.72 | ~53 | 75.24±9.61 | ~24 |
